# Integrative Proteomics Reveals That Ejiao Alleviates Premature Ovarian Insufficiency and Restores Bone Metabolism in Mice

**DOI:** 10.3390/nu18213595

**Published:** 2026-10-29

**Authors:** Changwei Wang, Xueyuan Fu, Qin Feng, Wanxiu Cao, Fen Du, Chuyi Liu, Xiaomei Feng, Shan Liu, Hu Hou, Bafang Li, Xiaoying Zhou

**Affiliations:** 1 Marine Biomedical Research Institute of Qingdao, Qingdao 266701, China; fuxueyuan@ouc.edu.cn (X.F.); caowanxiu2014@sina.com (W.C.); dufen0402@163.com (F.D.); liucy@ouc.edu.cn (C.L.); fxm1759@ouc.edu.cn (X.F.); liushan2007@126.com (S.L.); bfli@ouc.edu.cn (B.L.); 2 Shandong Key Laboratory of Gelatine Medicines Research and Development, Dong’e Ejiao Co., Ltd., No. 78, E-Jiao Street, Done-E Country, Liaocheng 252200, China; fengqin39@dongeejiao.com; 3 State Key Laboratory of Marine Food Processing & Safety Control, College of Food Science and Engineering, Ocean University of China, Qingdao 266000, China; houhu@ouc.edu.cn

**Keywords:** premature ovarian insufficiency, Ejiao, tripterygium glycosides, ovarian function, bone metabolism, proteomics, differentially expressed proteins

## Abstract

**Background:** Premature ovarian insufficiency (POI) is an intractable reproductive endocrine disease characterized by accelerated ovarian aging, accelerated follicle depletion and hormonal disorders, leading to secondary bone metabolic dysfunction and impaired long-term female health. Conventional hormone replacement therapy is the guideline-recommended first-line therapy for young women with POI, yet its long-term safety must be individualized; natural nutritional interventions are best viewed as complementary strategies. Donkey-hide gelatin (Ejiao), a natural food rich in bioactive collagen and amino acids, is commonly used for female reproductive care. Nevertheless, its systematic efficacy and molecular mechanisms against POI and related bone complications remain poorly defined. **Methods:** This study established a tripterygium glycosides (TG)-induced POI mouse model. Mice received low-dose (1.5 g/kg) and high-dose (3.0 g/kg) Ejiao treatment for 30 consecutive days. Reproductive organ morphology, estrous cycle, serum hormone levels, ovarian histopathology, bone microarchitecture and ovarian proteomic profiles were assessed. **Results:** Ejiao remarkably restored ovarian index and tended to improve uterine index (non-significant), corrected estrous cycle disturbance, and partially corrected the endocrine profile (E2, AMH, GnRH). It upregulated the ovarian reserve markers E2 and AMH and downregulated GnRH. Histological examination verified improved follicular development and reduced atresia. Furthermore, Ejiao alleviated POI-induced bone loss, optimized trabecular microstructure and rescued abnormal RANKL/OPG ratio to stabilize bone metabolism. Proteomic analysis revealed 313 differentially expressed proteins (DEPs) between Ejiao-treated and model groups, with enrichment in calcium signaling, cAMP, cGMP-PKG, and TGF-β/Smad pathways. **Conclusions:** As a natural bioactive food, Ejiao exerts dual protective effects against POI and concurrent bone metabolic dysfunction associated with modulation of multiple core signaling pathways and functional proteins (suggesting involvement of these pathways in its effects).

## 1. Introduction

Premature ovarian insufficiency (POI) is a heterogeneous reproductive endocrine disorder characterized by ovarian dysfunction in women under 40 years of age. It is mainly manifested as menstrual disturbance (oligomenorrhea or amenorrhea), hypoestrogenism, hypergonadotropinism, and impaired fertility [1]. Contrary to the traditional view of irreversible ovarian exhaustion, POI is now recognized as a variable condition with possible residual ovarian function. In recent years, the global incidence of POI has continued to rise and show a significant younger onset trend [2]. POI not only causes infertility but also leads to long-term complications including osteoporosis, cardiovascular diseases, and mental disorders, severely impacting women’s quality of life [3].

At present, hormone replacement therapy (HRT) is the guideline-recommended first-line treatment for young women with premature ovarian insufficiency (POI), effectively alleviating menopausal symptoms and protecting bone health [1,3,4]. However, the long-term safety profile of HRT must be individualized, and the Women’s Health Initiative (WHI) evidence—derived from older postmenopausal women—is not directly transferable to young POI patients [5]. Natural bioactive nutritional interventions, including Ejiao, should therefore be viewed as complementary strategies rather than categorically safer alternatives, and their role alongside or instead of HRT warrants further study.

Donkey-hide gelatin (Ejiao) is a unique animal-derived functional nutritional product in China with a 2000-year health application history [6]. It is rich in high-quality hydrolyzed collagen, essential amino acids, polypeptide active substances and trace mineral elements, and has unique nutritional advantages in nourishing reproductive tissues, regulating endocrine metabolism, improving tissue antioxidant capacity and repairing tissue damage [7]. Modern nutritional and pharmacological studies have confirmed that Ejiao can optimize the developmental status of female reproductive organs and regulate basic hormone levels. Existing studies have initially verified its regulatory effect on ovarian physiological function in aging mice [7,8,9,10]; nevertheless, systematic verification of its phenotypic intervention effect on POI and in-depth exploration of its molecular regulatory mechanism are still lacking, which greatly limits the modern nutritional popularization and industrial application of Ejiao in the field of female reproductive health.

The pathogenesis of POI involves complex molecular events including granulosa cell apoptosis, oxidative stress, chronic inflammation, and disruption of key signaling pathways that regulate follicular development and steroidogenesis. In particular, calcium signaling and cyclic nucleotide pathways (cAMP and cGMP-PKG) play essential roles in oocyte maturation, granulosa cell survival, and hormone synthesis [11,12,13,14]. The TGF-β/Smad signaling axis is critical for follicle activation, granulosa cell differentiation, and prevention of atresia [15,16]. Wnt/β-catenin signaling helps maintain the primordial follicle pool and regulates ovarian stromal function [17,18]. Furthermore, steroid hormone biosynthesis is the cornerstone of ovarian endocrine function, and its impairment directly leads to the hormonal imbalances seen in POI [19,20]. Dysregulation of extracellular matrix (ECM) remodeling by matrix metalloproteinases (MMPs), such as MMP14, has also been linked to follicular rupture defects and ovarian fibrosis [21,22].

The tripterygium glycosides (TG)-induced POI mouse model is a classic and mature chemical ovarian injury model, which can stably simulate the core pathological characteristics of clinical POI patients, including follicle over-atresia, hormonal disturbance, ovarian tissue damage and secondary bone loss, and is widely used in the efficacy evaluation and mechanism research of natural nutritional products for ovarian protection. In this study, TG-induced POI mice were used as the research object to systematically evaluate the intervention effect of Ejiao on POI macroscopic phenotypes including reproductive organ damage, estrous cycle disorder, endocrine imbalance, follicle loss and bone metabolic dysfunction. The results provide the first comprehensive evidence that Ejiao alleviates POI through multi pathway regulation, supporting its potential as a natural bioactive food for POI and its complications.

## 2. Materials and Methods

### 2.1. Materials and Reagents

Ejiao was provided by Shandong Dong-E-E-Jiao Co., Ltd. (Dong’e, China). Feed and bedding materials were obtained from Jinan Pengyue Experimental Animal Breeding Co., Ltd. (Jinan, China). Tripterygium glycosides for scientific research were purchased from Yuanda Pharmaceutical Huangshi Feiyun Pharmaceutical Co., Ltd. (Huangshi, China). Purple blood collection tubes (containing EDTA as an anticoagulant) and red blood collection tubes (anticoagulant-free) were supplied by Shandong Chengwu Yongkang Medical Products Co., Ltd. (Chengwu, China). Ophthalmic forceps were acquired from Xinhua Surgical Instrument Co., Ltd. (Zibo, China). Papanicolaou staining solution was obtained from Shanghai Yuanye Technology Co., Ltd. (Shanghai, China). Hematoxylin was purchased from Sigma-Aldrich (St. Louis, MO, USA). Water-soluble eosin Y, xylene, paraffin embedding agent, and neutral gum were obtained from Sinopharm Chemical Reagent Co., Ltd. (Beijing, China). Mouse follicle-stimulating hormone (FSH), estradiol (E2), luteinizing hormone (LH), anti-Müllerian hormone (AMH), Gonadotropin releasing hormone (GnRH) and prolactin (PRL) ELISA kits were purchased from Shanghai Sangon Biotech Co., Ltd. (Shanghai, China) and Jingmei Biological Engineering Co., Ltd. (Shanghai, China). All kits were used according to the manufacturers’ instructions. The intra-assay and inter-assay coefficients of variation were below 10% and 15%, respectively, ensuring high reproducibility and reliability of the hormonal measurements across experimental groups.

### 2.2. Animals and Experimental Design

Female ICR mice, aged 6–8 months with a body weight of 40 ± 2 g, were purchased from Jinan Pengyue Experimental Animal Breeding Co., Ltd. The animal production license number is SCXK(Lu) 2022 0006, and the animal quality certificate number is No.370726240101851856. The mice were housed in the animal room of the Functional Products Platform of Qingdao Marine Biomedical Research Institute. They were allowed to drink and eat freely (maximum 6 mice per cage), with a feeding temperature of (23 ± 2) °C, relative humidity of (50 ± 10)%, and a 12 h/12 h light-dark cycle. Their welfare was checked daily for activity, fur coat, body weight, food intake and signs of distress. After a week of adaptive feeding, the mice were randomly divided into four groups (*n* = 18 per group; 72 mice in total for the terminal endpoint cohort): a negative control group, a model group, an Ejiao low-dose group (1.5 g/kg/day), and an Ejiao high-dose group (3.0 g/kg/day).

Study design and ethical compliance: Mice were randomly allocated to the four groups using a computer-generated randomization sequence after stratification by baseline body weight. Investigators quantifying histology, immunohistochemistry, and micro-CT were blinded to group allocation. No animals were excluded or withdrawn. The exact number of animals per endpoint is reported in the relevant figure legends. This study complies with the ARRIVE 2.0 guidelines; a formal a priori power calculation was not performed, and sample sizes were based on established tripterygium-glycoside-induced POI literature.

To establish the premature ovarian insufficiency (POI) model, mice in the model group and the two Ejiao groups received tripterygium glycosides (50 mg/kg) by gavage daily [23]. Meanwhile, the negative control group was administered an equal amount of saline by gavage daily. To avoid repeated sampling stress interfering with terminal outcomes, serial hormonal monitoring was performed in a separate cohort of mice (*n* = 6 in total, repeatedly sampled at baseline, week 2, and week 4; 50 µL orbital blood per collection). These monitored animals were excluded from the final endpoint analyses. A priori model success criteria adapted from Chen et al. and Ma et al. were predefined before experimentation, including sustained elevation of serum FSH and LH, reduced serum E2 and AMH [24,25].

Following successful modeling, the negative control group and the model group continued to receive saline by gavage, while the two Ejiao groups received the respective doses (1.5 g/kg/day and 3.0 g/kg/day) by gavage for at least 30 consecutive days. The dosing regimen was derived using the FDA body surface area conversion factor of approximately 12.3. The low Ejiao dose (1.5 g/kg) was calculated by body surface area normalization and corresponds to a human daily dose within the clinically recommended range (3–9 g/day for a 60 kg adult), while the high dose (3.0 g/kg) was set as two times the low dose. Ejiao (batch No. 20231014) was supplied by Dong-E-E-Jiao Co., Ltd. Ejiao is a donkey-hide gelatin preparation whose protein fraction consists predominantly of type I collagen-derived gelatin and collagen-derived peptides, together with free amino acids. The manufacturer’s certificate of analysis reported a total protein content of 81.4%, a fat content of 6.38%, and a total sugar content of 7.65%. At the end of the treatment period, all mice were euthanized, and samples were collected for further analysis. All animal procedures were approved by the Ethics Committee of Qingdao Marine Biomedical Research Institute (approval No. E-MBGT-2024-11-12) and complied with relevant guidelines.

### 2.3. General Condition Observation and Estrous Cycle Monitoring

After the POI model was successfully established, vaginal smears were collected and examined every day for 11 consecutive days during the treatment period. The vaginal cavity of each mouse was gently flushed with physiological saline 5–6 times using a pipette, and the resulting cell suspension was evenly spread onto a glass slide [26]. After air drying, the smears were fixed with 95% ethanol for 15 min, then rehydrated through descending concentrations of ethanol (95%, 80%, and 70%) for 1 min each. The slides were stained with hematoxylin for 15 min, treated with blueing reagent for 1 min, and rinsed under tap water for 1–2 min. Subsequently, they were dipped in acid-alcohol (hydrochloric acid in ethanol) for 10 s, rinsed again with tap water for 5 min, dehydrated through ascending ethanol concentrations (70%, 80%, and 95%) for 1 min each, counterstained with eosin for 2 min, placed in 100% ethanol, air-dried, and mounted with neutral gum. The estrous cycle stage (proestrus, estrus, metestrus, diestrus) was determined for each mouse over the 11-day observation period according to the relative abundance and morphology of nucleated epithelial cells, cornified squamous epithelial cells, and leukocytes [27]. The rate of dysregulation of the estrous cycle in each group of mice is calculated as the number of dysregulated mice divided by the total number of mice in each group multiplied by 100%.

### 2.4. Sample Collection

No unexpected adverse events, deaths, or animals reaching humane endpoints (body weight loss > 20%, severe lethargy or inability to eat/drink) were observed throughout the experiment. After the last administration, mice were fasted overnight with free access to water. Animals were anesthetized via an intraperitoneal overdose of sodium pentobarbital (150 mg/kg), followed by cervical dislocation for rapid, painless euthanasia in accordance with AVMA guidelines. Orbital blood was collected using both EDTA-containing and non-anticoagulant tubes. Serum was separated by centrifugation (3000 rpm, 10 min) and stored at −80 °C for subsequent analysis. The ovaries, uterus, liver, kidneys, spleen, heart, and femurs were dissected, weighed for calculation of organ indices, and either stored at −80 °C or fixed in 4% paraformaldehyde.

### 2.5. Ovarian Histopathological Analysis

Ovarian follicle quantification was performed using 6 mice per group. Ovaries fixed in 4% paraformaldehyde were dehydrated, embedded in paraffin, and serially cut into 5-µm sections. Sections were stained with hematoxylin and eosin (H&E). Systematic sampling spanning the entire ovary from cortex to medulla was applied to minimize sampling bias; every fifth section was selected for analysis to avoid double counting, and follicles were counted only when the oocyte nucleus was visible. Follicle counting was performed by two independent blinded researchers under a light microscope. Follicles were systematically quantified and classified by developmental stage, including primordial and antral follicles, as well as primary (single layer of cuboidal cells), secondary (two or more layers, no antrum), atretic (degenerating oocyte or pyknotic nuclei), and corpus luteum [28], according to standard morphological criteria.

### 2.6. Serum Hormone and Factor Detection

At the end of the experiment, mice were euthanized and blood was collected (via retro-orbital bleeding following enucleation). To minimize circadian and cyclical variation, all blood samples were obtained at a fixed time of day (10:00 am), and all animals were sampled at the diestrus stage of the estrous cycle (confirmed by vaginal cytology). Serum was separated by centrifugation and stored at −80 °C until analysis, according to the manufacturers’ instructions. Serum concentrations of follicle-stimulating hormone (FSH), luteinizing hormone (LH), estradiol (E2), anti-Müllerian hormone (AMH), prolactin (PRL), and gonadotropin-releasing hormone (GnRH) were measured in duplicate using commercially available ELISA kits according to the manufacturers’ protocols.

### 2.7. Bone Metabolism Evaluation

Flow cytometry: Peripheral blood mononuclear cells (PBMCs) were isolated from peripheral blood using lymphocyte separation medium, and dead cells were excluded using a fixable viability stain 510 (BD Pharmingen, cat. no. 564406). Cells were surface-stained with FITC-conjugated anti-CD4 (BD Pharmingen, cat. no. 561835) and APC-conjugated anti-CD25 (BD Pharmingen, cat. no. 561048); after fixation and permeabilization, cells were stained with PE-conjugated anti-Foxp3 (BD Pharmingen, cat. no.560414). Compensation was performed using single-stained compensation beads. For each sample, ≥10,000 events were acquired on a CytoFLEX flow cytometer (Beckman Coulter, Brea, CA, USA) and analyzed using CytExpert software (Version 2.4, Beckman Coulter). After fixation and permeabilization for intracellular staining, the proportion of CD4^+^CD25^+^Foxp3^+^ regulatory T cells (Tregs) was determined by flow cytometry [29].

Immunohistochemistry was performed on femoral bone tissue. Femur samples from 6 animals per group were decalcified, embedded in paraffin and cut into 4-µm-thick serial sections. Sections were then incubated with primary antibodies against OPG (anti-osteoprotegerin antibody, ab73400; Abcam, Cambridge, UK) and RANKL (anti-RANKL antibody, ab216484; Abcam, Cambridge, UK), followed by detection using an Elivision plus immunohistochemistry detection kit (Fuzhou Maixin Biotech Development Co., Ltd., Fuzhou, China). For each section, randomly selected fields at ×200 magnification were captured, and the average optical density of the positive staining was analyzed using Image-Pro Plus 6.0 software (Media Cybernetics, Inc., Rockville, MD, USA). Quantification was performed by an investigator blinded to the experimental groups. OPG and RANKL immunoreactivity in bone tissue was thereby evaluated [30].

Bone microarchitecture was assessed by micro-computed tomography (micro-CT). The distal femur of each mouse was scanned using a Quantum GX2 micro-CT imaging system (Revvity, Inc., Waltham, MA, USA) at an isotropic voxel size of 10 µm, with an X-ray tube voltage of 50 kV and a current of 100 µA. Images were reconstructed using the Quantum GX imaging system software (Quantum GX Analysis Software, v2.2, Revvity, Inc., Waltham, MA, USA). A three-dimensional region of interest (ROI) of 0.9 mm, spanning 0.1–1.0 mm above the growth plate of the distal femur, was selected for analysis. Bone mineral density (BMD) was calibrated using hydroxyapatite phantoms with known densities. From the reconstructed three-dimensional images, the following trabecular parameters were calculated using the system’s analysis software: bone mineral density (BMD), bone surface fraction (BS/TV), bone surface/bone volume ratio (BS/BV), trabecular number (Tb.N), trabecular thickness (Tb.Th), and trabecular separation (Tb.Sp) [31,32].

### 2.8. Proteomics Analysis

Sample Handling: Quantitative proteomics was performed on three pooled biological replicates per group; each pooled replicate consisted of ovaries pooled from three mice (i.e., three mice per pool, nine mice contributing per group). The effective proteomic sample size is therefore *n* = 3 pooled replicates per group, and all inferential statistics for proteomics are conducted at the pool level, not as nine independent observations. Each pooled sample was analyzed once by mass spectrometry. The groups were negative control group, model group and Ejiao group (low-dose Ejiao group). Begin by retrieving the samples from the −80 °C freezer and grinding them into powder using liquid nitrogen. Next, transfer an appropriate amount of powder to 1.5 mL centrifuge tubes, add lysis buffer (containing 8 M urea, 1 mM PMSF, and 2 mM EDTA), perform ultrasonic lysis for 5 min on ice, and centrifuge at 15,000× *g* at 4 °C for 10 min to collect the supernatant. Finally, determine the protein concentration using a BCA assay kit.

Proteolytic Desalting: Start by taking 100 μg of protein solution based on protein concentration, then adjust the volume to 200 μL with 8 M urea, followed by reduction with 5 mM DTT at 37 °C for 45 min, and alkylation with 11 mM iodoacetamide (IAM) in a dark room at room temperature for 15 min. Then, add 800 μL of 25 mM ammonium bicarbonate solution and 3 μL of Trypsin (Promega), and digest overnight at 37 °C. Adjust the pH of the digested peptides to 2–3 using 20%TFA, followed by desalting with C18 (Millipore, Billerica, MA, USA) resin. Finally, determine the peptide concentration using a Pierce™ Quantitative Peptide Assay Kit with standards (Thermo Fisher, Waltham, MA, USA) [33].

LC-MS/MS Analysis:

Liquid Chromatography Detection: Samples were separated using the Vanquish Neo UHPLC liquid chromatography system. The mobile phase A consisted of 0.1% formic acid aqueous solution, while mobile phase B was acetonitrile containing 0.1% formic acid. The injection mode employed a trap-and-elute dual-column method, with a PepMap Neo Trap Cartridge (300 μm × 5 mm, 5 μm) as the trapping column and an Easy-Spray™ PepMap™ Neo UHPLC column (150 μm × 15 cm, 2 μm) as the analytical column. The column temperature was controlled at 55 °C, with an injection volume of 200 ng, a flow rate of 2.5 μL/min, an effective gradient of 6.9 min, and a total runtime of 8 min [34,35].

Orbitrap Astral Mass Spectrometry Detection: DIA analysis utilized the Vanquish Neo system (Thermo Fisher Scientific) for chromatographic separation. Samples separated by nano-flow high-performance liquid chromatography were subjected to DIA (Data-Independent Acquisition) mass spectrometry analysis using the Orbitrap Astral high-resolution mass spectrometer (Thermo Scientific). The detection mode was positive ion mode, with a precursor ion scan range of 380–980 *m*/*z*, a primary mass resolution of 240,000 at 200 *m*/*z*, a Normalized AGC Target of 500%, and a Maximum IT of 5 ms. MS2 was performed using DIA data acquisition mode, with 299 scan windows, an Isolation Window of 2 Th, HCD Collision Energy of 25%, a Normalized AGC Target of 500%, and a Maximum IT of 3 ms.

Database search and quantification: MS raw data were analyzed using DIA-NN (v1.8.1) with a library-free approach. The UniProtKB mouse proteome database (UP000000589, containing 54,910 sequences) was used to generate a spectral library via deep learning-based algorithms. The “Match Between Runs” (MBR) option was enabled to build a spectral library from DIA data, and then the data were re-analyzed using this library. The false discovery rate (FDR) was set to <1% at both the protein and precursor ion levels. Only identifications meeting this threshold were retained for further quantification [36,37,38].

For quantitative data processing, proteins with >30% missing values across samples were excluded, and the remaining missing values were imputed using the minimum-value (left-censoring) approach. Data were then normalized using median/quantile normalization. Quality control was performed on pooled QC samples, with an acceptance criterion of a coefficient of variation (CV) < 20%. Differentially expressed proteins (DEPs) were defined as follows: for two-group comparisons, proteins with fold change (FC) ≥ 1.5 or FC ≤ 0.6667 and *p* ≤ 0.05 were considered significantly different; for comparisons involving more than two groups, *p* ≤ 0.05 was required.

Bioinformatics analyses were performed using DIA-NN (v1.8.1), R (v4.2.2) with the clusterProfiler package (v4.2.2), and STRING (v12). Gene Ontology (GO) enrichment analysis of the DEPs was performed across the three categories—biological process (BP), cellular component (CC), and molecular function (MF)—and Kyoto Encyclopedia of Genes and Genomes (KEGG) pathway enrichment was performed using clusterProfiler. The enrichment background comprised all quantified proteins, and significance was defined as adjusted *p* < 0.05 after Benjamini–Hochberg correction. Protein–protein interaction (PPI) networks were constructed using STRING (v12) with a confidence threshold of ≥0.7 and visualized in Cytoscape (v3.9.1, Cytoscape Consortium, San Diego, CA, USA); hub proteins were defined as those with a degree of ≥8 (top~5% of connectivity).

### 2.9. Statistical Analysis

Data were analyzed using GraphPad Prism 9.0 and expressed as mean ± standard deviation (x ± s). Longitudinal outcomes (body weight, serial hormone measurements) were analyzed by two-way repeated-measures ANOVA or linear mixed models, while cross-sectional endpoints were analyzed by one-way ANOVA followed by Tukey’s post-hoc test. Normality (Shapiro–Wilk) and homogeneity of variance (Levene) were assessed. effect sizes (η^2^) and 95% confidence intervals are reported alongside *p* values. A significance level of *p* < 0.05 was considered statistically significant (* *p* < 0.05, ** *p* < 0.01, *** *p* < 0.001, **** *p* < 0.0001). Tukey’s test controls the comparison-wise error rate within a family of comparisons but not across families; conclusions for exploratory endpoints are tempered accordingly, and Benjamini–Hochberg correction was applied where indicated. All measurements were performed using at least three biological replicates per group for every outcome parameter.

## 3. Results

### 3.1. Body Weight Changes and Organ Indices

**Figure 1:**
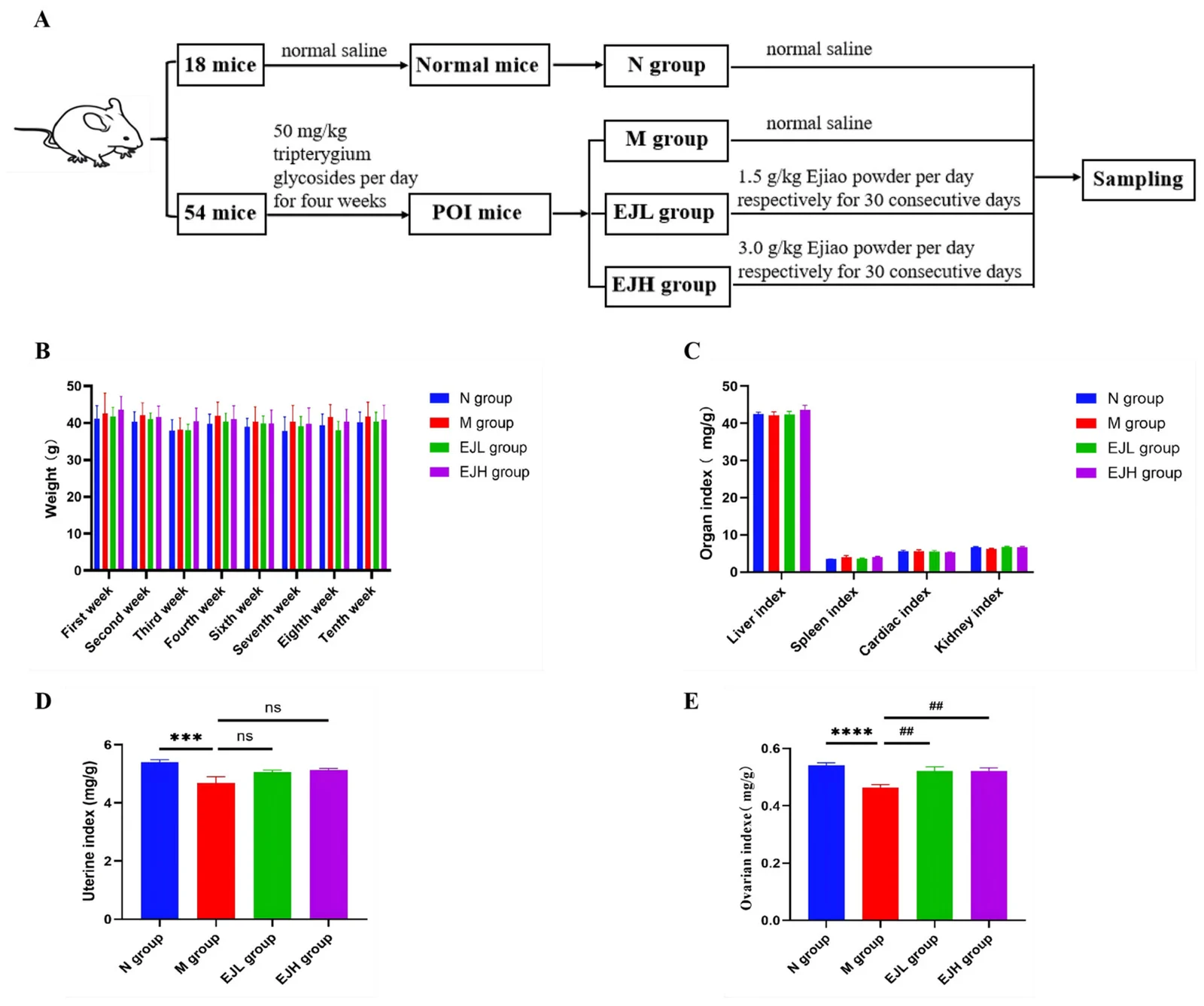
Experimental design and effects of Ejiao on body weight and visceral tissues in POI mice. (**A**) Experimental design; (**B**) Body weight; (**C**) Visceral indices of liver, spleen, cardiac and kidney; (**D**) Uterine index; (**E**) Ovarian index. Statistical significance is indicated as follows: ns (not significant) *p* > 0.05, ## *p* < 0.01, *** *p* < 0.001, **** *p* < 0.0001. (*n* = 10 mice per group).

In this study, there were no significant differences in body weight, liver index, spleen index, cardiac index, or kidney index among the negative control group, model group, and Ejiao dose groups (Figure 1B,C). Compared with the negative control group, the model group showed a significant decrease in both uterine index and ovarian index (*p* < 0.001 or *p* < 0.0001). In contrast, treatment with Ejiao significantly increased the ovarian index compared with the POI group (*p* < 0.01) (Figure 1E), while a tendency toward improvement in the uterine index was observed, though without statistical significance (Figure 1D). These results provide preliminary evidence that Ejiao may ameliorate tripterygium glycosides-induced POI, consistent with previous findings that TG exposure leads to reduced ovarian and uterine index in mice [24]. Notably, comparable effects of other nutraceuticals on ovarian/uterine indices have been reported (see Discussion for a cross-comparison).

### 3.2. Effect of Ejiao on Estrous Cycle of POI Mice

Based on the characteristics of vaginal cell changes, the levels of estrous cycle disorder (Grade I–IV) are classified as follows: Grade I: normal estrous cycle; Grade II: regular estrous cycle with shortened estrus phase; Grade III: irregular estrous cycle accompanied by prolonged diestrus phase, with prolonged or normal estrus phase; Grade IV: absence of cyclicity. A well-established POI animal model should recapitulate the pathological features of human POI, reflecting ovarian damage induced by POI, including altered estrous cycles, significantly changed serum sex hormone levels, and markedly reduced follicle numbers [24].

**Figure 2:**
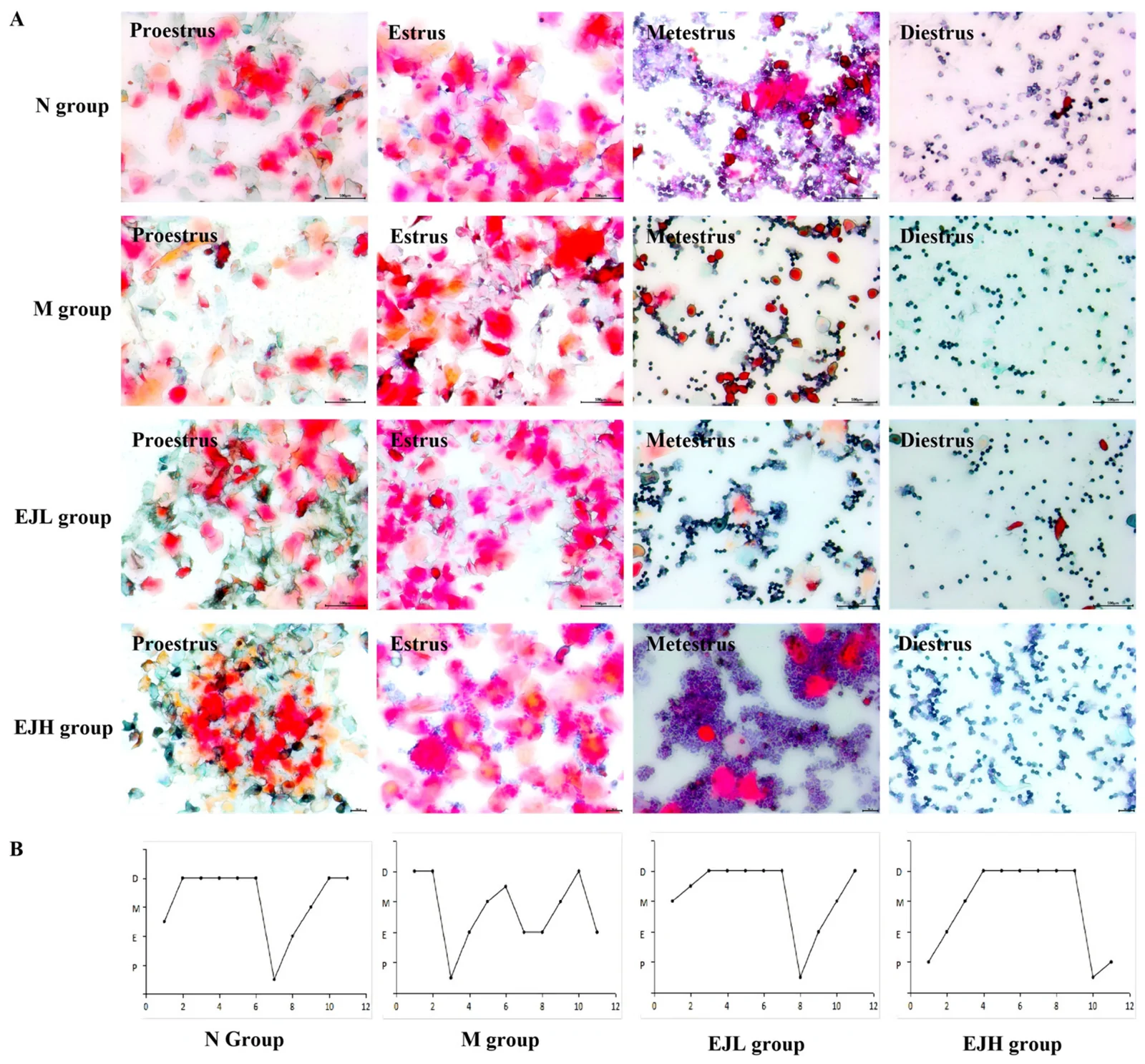
The effect of Ejiao on the estrus cycle. (**A**) Stained smears representing the phases of the estrous cycle: proestrus, estrus, metestrus, and diestrus; (**B**) Pattern diagram of estrous cycles in different groups. Note: The cycle is divided into four stages: proestrus (P), estrus (E), metestrus (M), and diestrus (D). (*n* = 6 mice per group).

Estrous cycle length was defined as the interval between consecutive estrus phases or sequential proestrus–estrus events. After continuous monitoring for 11 consecutive days (at 24-h intervals), the average estrous cycle length and the number of mice at each grade of estrous cycle dysfunction (Grade I–IV) are listed in Table 1. Mice in the negative control group showed regular cyclic alternation of proestrus, estrus, metestrus and diestrus, with a mean cycle length of 9.3 days, and all six mice remained at Grade I. In contrast, the model group exhibited severe estrous cycle disruption, with a significantly prolonged average cycle length of 10.8 days; most animals (*n* = 4) developed Grade III dysfunction. These findings confirm that tripterygium glycosides disrupt physiological vaginal cyclic changes, induce estrous cycle disorder and cause ovarian dysfunction [24]. Compared with the model group, Ejiao treatment reduced estrous cycle duration, with mean cycle lengths of 9.8 days (EJL, low dose) and 8.5 days (EJH, high dose). Three mice in both treatment groups recovered to Grade I normal cyclic status. Full monitoring data are provided in Table 1, and representative estrous cycle patterns and vaginal exfoliated cell images are shown in Figure 2.

### 3.3. Effect of Ejiao on Tissue Structure of Ovary in POI Mice

**Figure 3:**
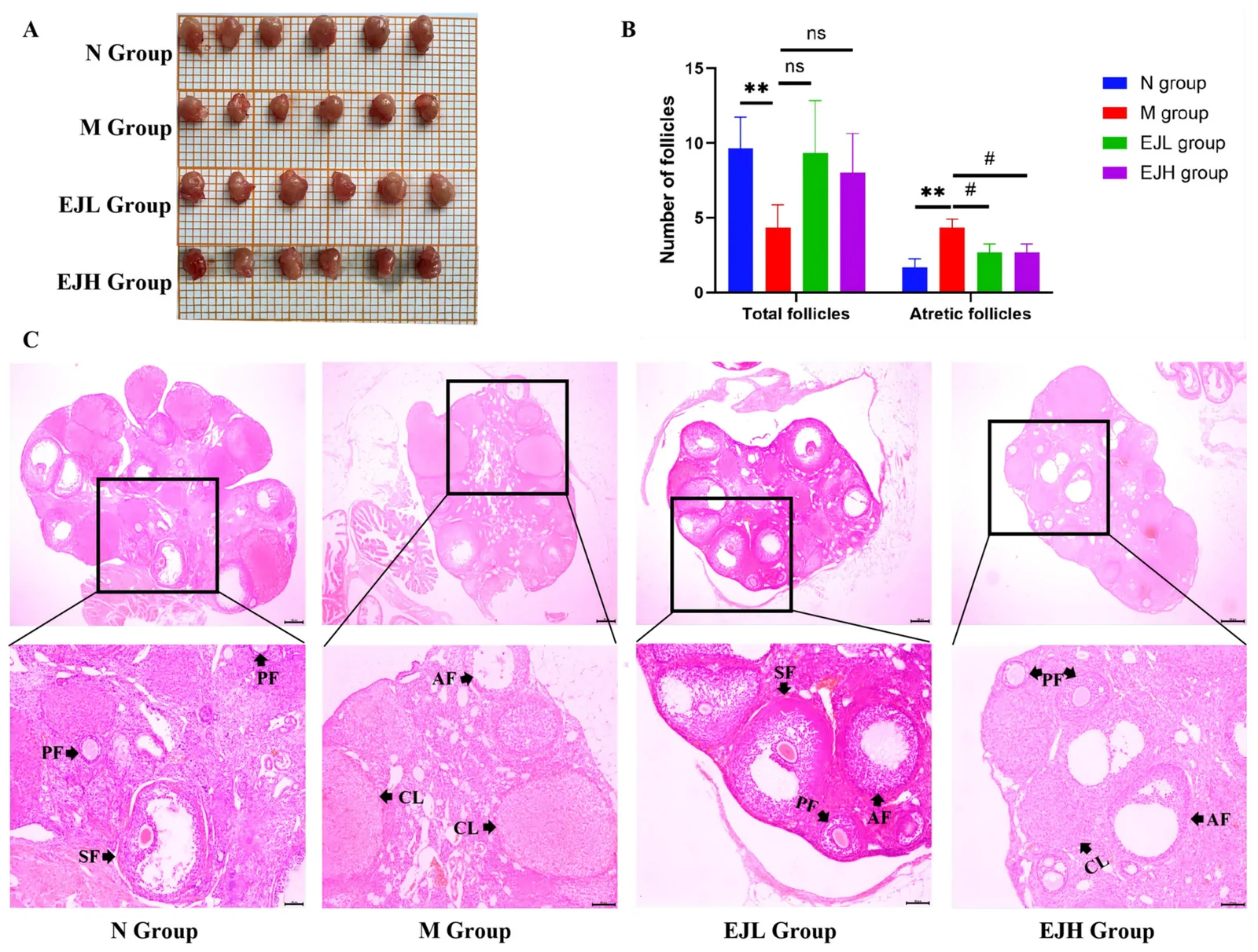
Ejiao improved ovarian weight ratio and follicular development in POI mice. (**A**): Ovarian tissues from six mice per treatment group were displayed (scale bar: 1 cm). (**B**,**C**): After HE staining, the structure of follicles at all levels was observed under a 40× and 100× microscope, and the number of follicles in different stage (Primary follicle (PF), Secondary follicle (SF), Atresia follicle (AF), Corpus Luteum (CL)) was counted by tissue sections (Arrows indicate oocytes at different developmental stages). (ns *p* > 0.05, ** *p* < 0.01, # *p* < 0.05). (*n* = 6 mice per group).

Ejiao improves ovarian weight ratio and follicular development in tripterygium glycosides-induced POI Mice. As described previously (Section 2.2), ovaries were harvested from POI model mice after intragastric administration of Ejiao for 30 days. Figure 3A shows ovarian atrophy in POI mice; by contrast, ovarian volume and weight were restored in Ejiao-treated groups (*p* < 0.01), consistent with the aforementioned ovarian index data (Figure 1E).

As shown in Figure 3B,C, ovaries in the negative control group exhibited clearly defined growing follicles at all developmental stages, with densely packed granulosa cell layers surrounding the follicles; the numbers of total follicles and atretic follicles were normal. Ovarian tissues in the model group showed structural damage, decreased numbers of follicles at each stage, significantly reduced total follicle count, and markedly increased atretic follicles with no dominant follicles (*p* < 0.01), indicating degenerative follicular development and severe ovarian dysfunction, which confirmed successful model establishment [24]. Compared with the M group, pathological ovarian lesions were alleviated in the low-dose Ejiao (EJL) and high-dose Ejiao (EJH) groups. The number of follicles at each stage increased while atretic follicles decreased significantly (*p* < 0.05), accompanied by more regularly arranged granulosa cell layers. These findings demonstrate that Ejiao ameliorates abnormal follicular development in POI mice. (see Discussion for comparison with other nutraceuticals).

### 3.4. Effect of Ejiao on Reproductive Hormone Level in POI Mice

Premature ovarian insufficiency (POI) is closely associated with hormonal alterations, clinically characterized by hypoestrogenemia and hypergonadotropinemia. Among sex hormones, follicle-stimulating hormone (FSH), luteinizing hormone (LH) and prolactin (PRL) are secreted by the pituitary gland, whereas estradiol (E2) and anti-Müllerian hormone (AMH) are mainly produced by the ovary [39,40]. Gonadotropin-releasing hormone (GnRH), a glycoprotein hormone that regulates gonadal development and stimulates sex hormone synthesis and secretion in vertebrates, is predominantly synthesized and released by GnRH-secreting neurons in the hypothalamus [41]. These hormones serve as core biomarkers reflecting ovarian reserve, follicular function, and homeostasis of the hypothalamic-pituitary-ovarian (HPO) axis.

**Figure 4:**
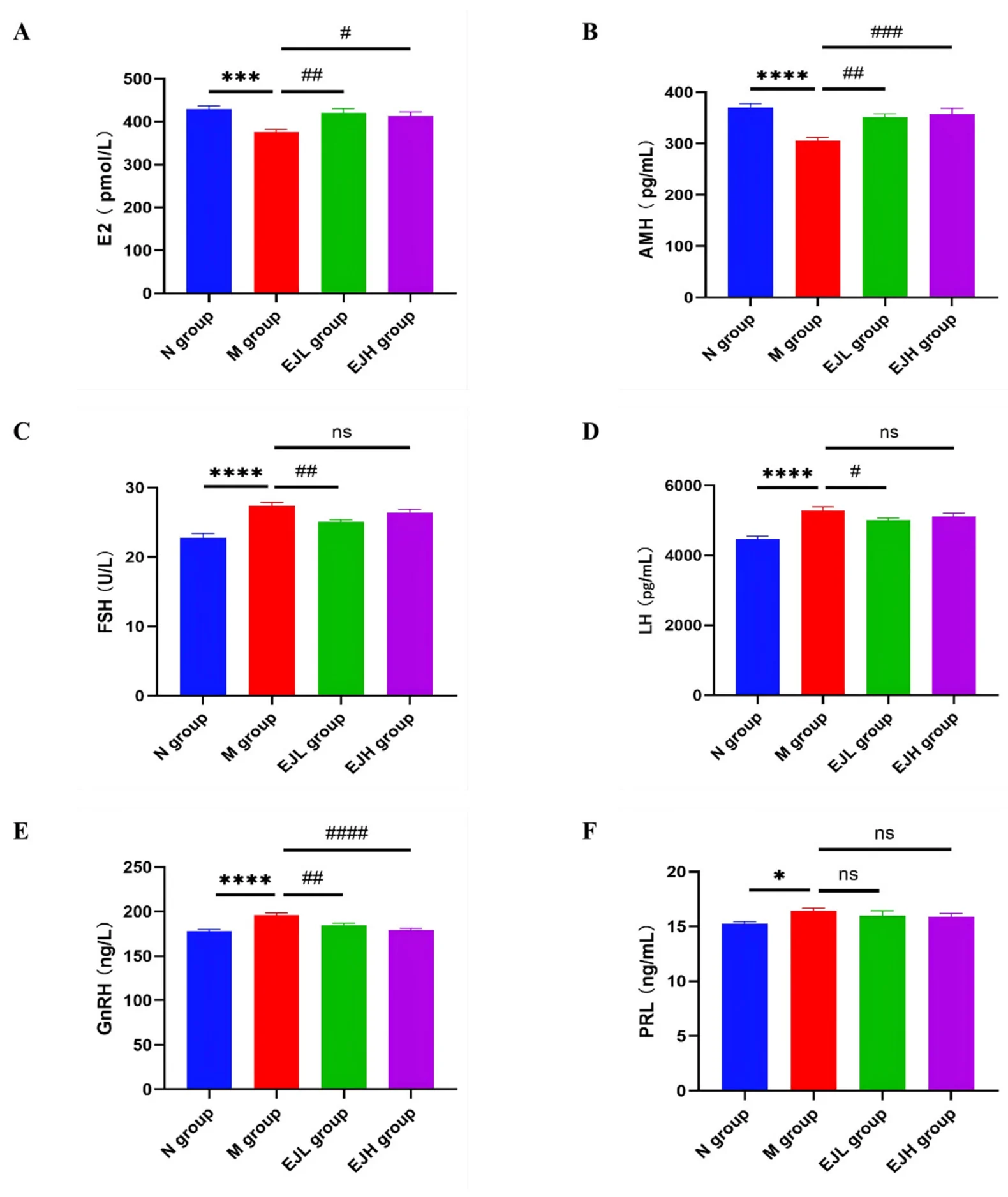
Effect of Ejiao on reproductive hormone level in POI mice. (**A**) E2; (**B**) AMH; (**C**) FSH; (**D**) LH; (**E**) GnRH; (**F**) PRL. Statistical significance is indicated as follows: ns *p* > 0.05, *,# *p* < 0.05, ## *p* < 0.01, ***,### *p* < 0.001, and ****,#### *p* < 0.0001. (*n* = 10 mice per group).

In this study, six key serum reproductive hormones (E2, AMH, FSH, LH, GnRH and PRL) were measured to further investigate the regulatory effect of Ejiao on endocrine homeostasis in POI mice (Figure 4). The negative control group (N group) exhibited stable physiological levels of all detected hormones, with high concentrations of E2 and AMH and low basal levels of FSH, LH, GnRH and PRL, indicating intact ovarian reserve and normal HPO-axis function.

In contrast, the tripterygium glycosides-induced POI model group (M group) exhibited marked endocrine dysregulation. Compared with the N group, serum E2 and AMH levels were significantly decreased (*p* < 0.001 or *p* < 0.0001), while the concentrations of FSH, LH, GnRH and PRL were significantly increased (*p* < 0.0001 or *p* < 0.05). These typical hormonal changes are consistent with the clinical manifestations of human POI, verifying the successful establishment of a POI mouse model with impaired ovarian function and disrupted HPO-axis secretion.

Following Ejiao intervention, compared with the POI model group (Group M), serum E2 and AMH concentrations were significantly elevated in both low-dose and high-dose Ejiao groups (*p* < 0.01 or *p* < 0.001), whereas GnRH levels were markedly reduced (*p* < 0.01 or *p* < 0.0001). FSH and LH concentrations were significantly decreased in the low-dose Ejiao group (*p* < 0.05 or *p* < 0.01), while no significant reduction was observed in the high-dose Ejiao group. No significant differences in PRL levels were detected in either low- or high-dose Ejiao groups (*p* > 0.05).

Collectively, these findings suggest that Ejiao ameliorates POI-associated endocrine disturbances by increasing E2 and AMH and modulating the secretion of GnRH, FSH, and LH, thereby improving HPO axis homeostasis and preserving ovarian function in POI mice.

### 3.5. Effect of Ejiao on Bone Metabolism

**Figure 5:**
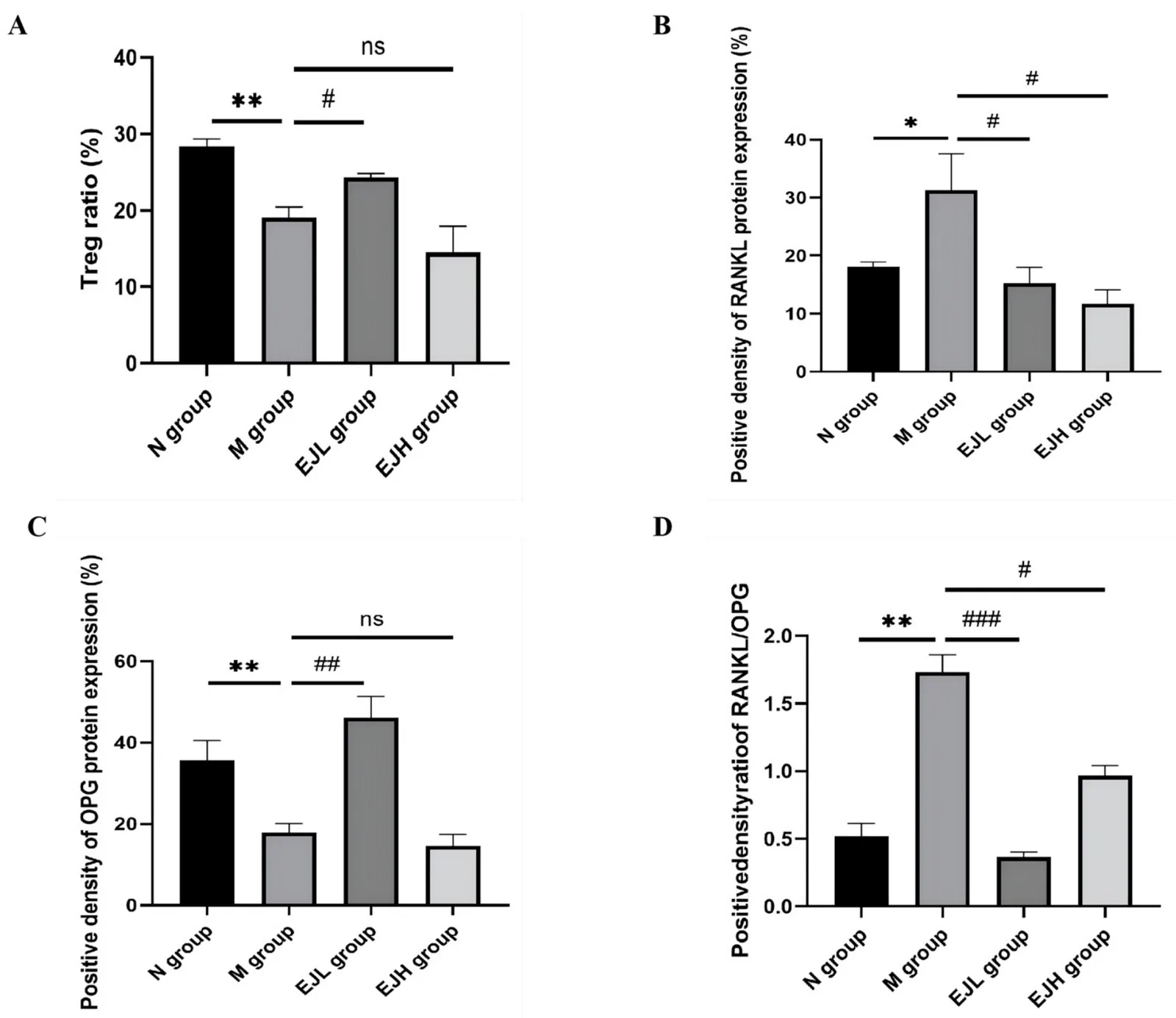
Ejiao modulates Treg proportion and bone RANKL/OPG immunohistochemistry expression in POI mice. (**A**) Treg ratio; (**B**) RANKL; (**C**) OPG; (**D**) RANKL/OPG. Statistical significance is indicated as follows: ns *p* > 0.05, *,# *p* < 0.05, **,## *p* < 0.01, ### *p* < 0.001. (*n* = 6 mice per group).

Clinically, estrogen deficiency in patients with premature ovarian insufficiency (POI) causes reduced bone mass and altered bone microarchitecture, ultimately leading to osteoporosis [42]. Studies have demonstrated that peripheral regulatory T (Treg) cells promote bone repair and regeneration, and are essential for maintaining bone homeostasis [43]. Specifically, Treg cells are an immunosuppressive T-cell subset that modulate systemic immune responses and shift pro-inflammatory status toward an anti-inflammatory phenotype via secreting signaling molecules. Direct injection of Treg cells into injured bone has been reported to significantly accelerate bone healing [44]. In this study, flow cytometry was applied to analyze the proportion of peripheral blood Treg cells in mice across different groups. The results showed that the percentage of peripheral Treg cells was significantly decreased in the model group compared with the negative control group (*p* < 0.05). Relative to the model group, the proportion of peripheral Treg cells was markedly elevated in the low-dose Ejiao group (*p* < 0.05), whereas no obvious change was observed in the high-dose Ejiao group (Figure 5A).

Furthermore, the RANKL/OPG signaling axis acts as a pivotal pathway regulating osteoclast differentiation and maturation, which plays a vital role in bone resorption and remodeling [45]. RANKL is a key regulator of osteoclast proliferation, differentiation and activation; it binds to its receptor to induce differentiation and maturation of osteoclast precursors, thereby mediating bone resorption. In contrast, osteoprotegerin (2) functions as an inhibitor of osteoclast maturation and activation. It competitively binds to RANKL to suppress osteoclast differentiation and activity. OPG deficiency has been proven to aggravate bone remodeling dysfunction and osteoporosis [46]. Herein, POI model mice were used to investigate the effects of Ejiao on the expression of these two proteins in bone tissue.

Our results revealed that compared with the negative control group, OPG expression was significantly down-regulated in bone tissues of POI mice (*p* < 0.01), RANKL expression was significantly up-regulated (*p* < 0.05), and the RANKL/OPG ratio was markedly increased (*p* < 0.01), indicating excessive proliferation and differentiation of osteoclasts under POI conditions. After intragastric administration of Ejiao, OPG expression was significantly increased in the low-dose Ejiao group (*p* < 0.01) and RANKL expression was markedly decreased (*p* < 0.05). Moreover, the RANKL/OPG ratio was significantly reduced in both low- and high-dose Ejiao groups (*p* < 0.01 or *p* < 0.001) (Figure 5B–D). These findings align with previous reports showing that Ejiao enhances osteoblast differentiation by increasing alkaline phosphatase synthesis and reducing bone remodeling markers in ovariectomised rats [47]. Collectively, these results suggest that Ejiao inhibits bone resorption by down-regulating the RANKL/OPG ratio, thereby improving abnormal bone mineral density and bone microarchitecture in POI model mice.

**Figure 6:**
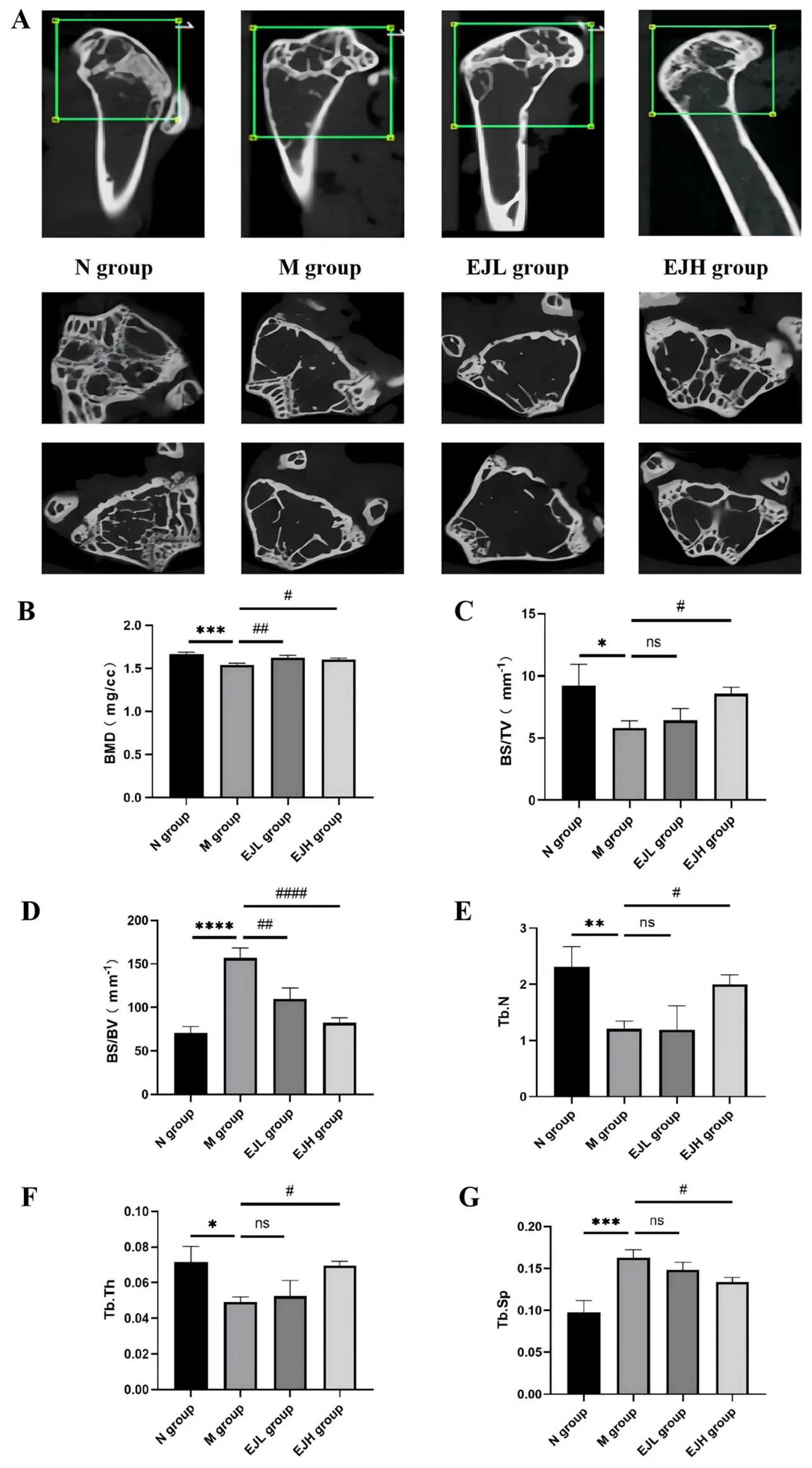
Ejiao ameliorates bone microarchitectural deterioration in POI mice. (**A**) Micro-CT images; (**B**) BMD; (**C**) BS/TV; (**D**) BS/BV; (**E**) Tb.N; (**F**) Tb.Th; (**G**) Tb.Sp. Statistical significance is indicated as follows: ns *p* > 0.05, *,# *p* < 0.05, **,## *p* < 0.01, *** *p* < 0.001, and ****,#### *p* < 0.0001. (*n* = 6 mice per group).

Micro-computed tomography (micro-CT) analysis was performed to evaluate bone microstructure and bone mineral density (BMD) in the femur of tripterygium glycosides-induced POI mice (Figure 6). Representative two-dimensional sagittal and transverse micro-CT images of femoral trabecular bone are displayed in Figure 6A. Distinct trabecular bone loss, sparse trabecular network, and widened medullary cavity were observed in the model (M) group compared with the normal control (N) group, whereas these pathological alterations were markedly rescued after Ejiao intervention.

Quantitative bone morphometric parameters are summarized in Figure 6B–G. Compared with the N group, the M group exhibited significantly decreased BMD (*p* < 0.001), BS/TV (*p* < 0.05), Tb.N (*p* < 0.01) and Tb.Th (*p* < 0.05), accompanied by notably increased BS/BV (*p* < 0.0001) and Tb.Sp (*p* < 0.001). These changes confirmed severe trabecular bone deterioration, reduced bone mass, and disrupted bone microarchitecture in POI mice, consistent with clinical osteoporosis manifestations in POI patients [48].

After treatment with low-dose (EJL) and high-dose (EJH) Ejiao, bone microarchitectural parameters were significantly improved. Relative to the M group, BMD, BS/TV, Tb.N and Tb.Th were significantly elevated (*p* < 0.05), while BS/BV and Tb.Sp were remarkably decreased (*p* < 0.05). Specifically, the EJH group showed superior protective effects on trabecular bone reconstruction compared with the EJL group, with more compact trabecular structure and higher trabecular number. No significant differences were found in partial indicators between EJL and EJH groups (ns).

Taken together, these micro-CT results demonstrate that Ejiao effectively mitigates POI-induced bone loss and trabecular microstructural damage by increasing bone density, improving trabecular connectivity, and reducing bone resorption, thereby exerting anti-osteoporotic effects in POI mice.

### 3.6. Results of Proteomic Analysis

#### 3.6.1. Proteomic Profiling Reveals Reversal of Ovarian Protein Expression by Ejiao

**Figure 7:**
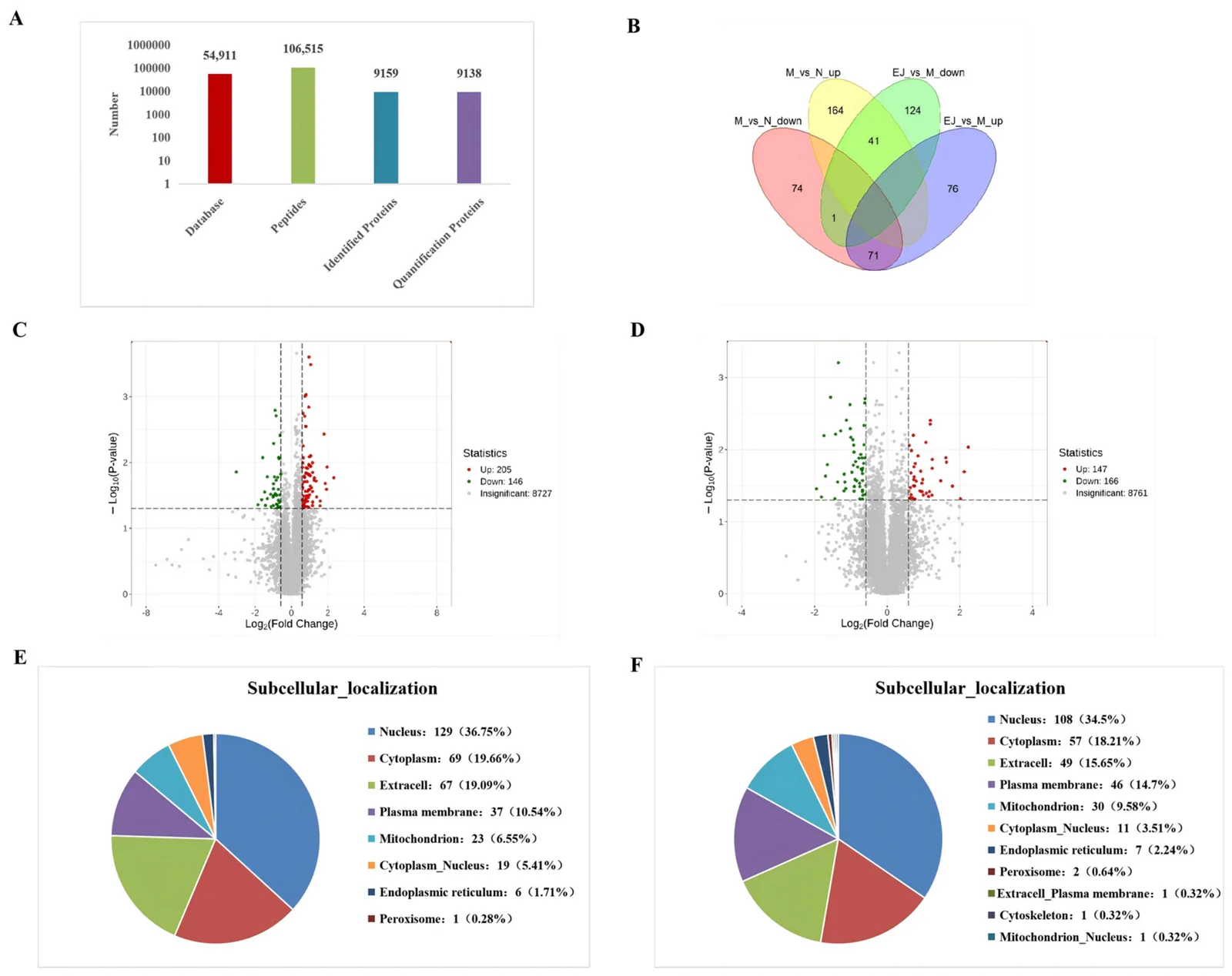
Differentially expressed proteins (DEPs) between different groups were identified via quantitative proteomics. (**A**) Basic statistical results of mass spectrometry data. (**B**) Venn diagram of DEPs. (**C**,**D**) Volcano plots of DEPs for the model-versus-normal control group and the Ejiao-versus-model group. Red dots represent up-regulated proteins, and green dots represent down-regulated proteins (The vertical dashed lines correspond to fold change; the horizontal dashed line indicates the statistical significance threshold of *p* < 0.05). (**E**,**F**) Subcellular localization of DEPs for the model-versus-normal control group and the Ejiao-versus-model group. [*n* = 3 biological replicates per group (each pool = 3 mice; 9 mice contributed per group).]

To explore molecular alterations associated with Ejiao intervention in a premature ovarian insufficiency (POI) model, data-independent acquisition (DIA)-based quantitative proteomics was performed on ovarian tissues from the normal control (N), POI model (M), and Ejiao-treated (EJ) groups. Basic statistical information of the proteomic dataset, including the total number of identified proteins across all groups, is shown in Figure 7A. A total of 351 differentially expressed proteins (DEPs) were identified between the M and N groups (205 upregulated, 146 downregulated), and 313 DEPs were identified between the EJ and M groups (147 upregulated, 166 downregulated) (Figure 7B). Venn diagram analysis showed that 41 proteins were significantly upregulated in the model group and subsequently downregulated after Ejiao intervention, while 71 proteins were significantly downregulated in the model group and upregulated after Ejiao treatment. These reversibly regulated proteins represent candidate core mediators of the therapeutic effects of Ejiao against POI (Figure 7B). Volcano plots further confirmed that POI induction caused extensive disruption of the ovarian protein expression profile, whereas Ejiao intervention effectively reversed this proteomic imbalance, gradually restoring abnormal protein expression toward normal physiological levels (Figure 7C,D).

Subcellular localization analysis was performed to characterize the distribution of differentially expressed proteins (DEPs) in the M vs. N (POI model vs. control) and EJ vs. M (Ejiao treatment vs. model) comparisons (Figure 7E,F). In both comparisons, DEPs were predominantly localized to the nucleus, cytoplasm, and plasma membrane, followed by the extracellular space, mitochondria, and cytoskeleton. In the M vs. N comparison, nuclear proteins accounted for 36.75%, cytoplasmic proteins for 19.66%, extracellular proteins for 19.09%, and plasma membrane proteins for 10.54%. After Ejiao treatment, the proportion of nuclear proteins decreased slightly to 34.5%, whereas plasma membrane-associated proteins increased markedly to 14.7%, and mitochondrial proteins rose from 6.55% to 9.58%. This redistribution suggests that Ejiao may help restore cellular compartment homeostasis by enhancing membrane-associated and mitochondrial functions while attenuating aberrant nuclear and extracellular signals, suggesting altered compartmental distribution that warrants functional validation.

#### 3.6.2. Pathway Enrichment Highlights TGF β/Smad and Wnt/β Catenin as Key Mediators

**Figure 8:**
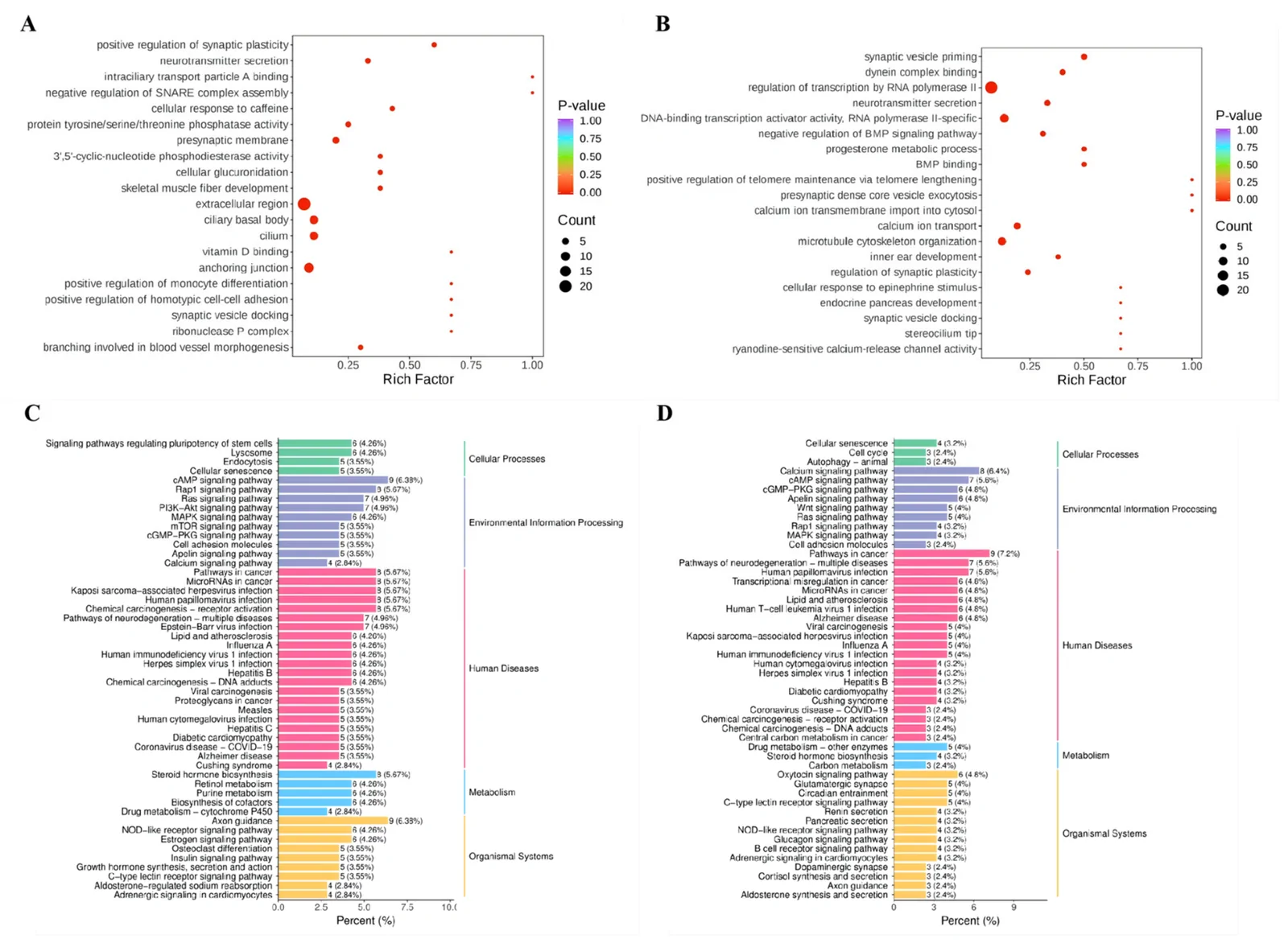
Perform bioinformatics analysis on DEP through GO and KEGG enrichment. (**A**,**B**) GO enrichment analysis bubble chart of DEPs. (**C**,**D**) KEGG classification bar chart of DEPs. [*n* = 3 biological replicates per group (each pool = 3 mice; 9 mice contributed per group).]

To characterize the biological functions and molecular features of differentially expressed proteins (DEPs), Gene Ontology (GO) and KEGG pathway enrichment analyses were performed (Figure 8).

GO enrichment (Figure 8A,B) revealed that, in the M_vs_N network (M_vs_N), the most prominent biological processes involved transcription regulation (RNA polymerase II, 16 proteins), extracellular matrix components (22 proteins), anchoring junctions, cilia, and vitamin D binding. Highly enriched terms with strong significance included positive regulation of monocyte differentiation, anchoring junctions, ciliary basal bodies, and synaptic plasticity, suggesting aberrant immune differentiation, defective cell–cell adhesion, ciliary dysfunction, and disturbed neuroendocrine crosstalk in the ovarian microenvironment. In contrast, the EJ_vs_M network (EJ_vs_M) shifted enrichment toward processes linked to ovarian function recovery, including microtubule cytoskeleton organization (8 proteins), calcium ion transport (5 proteins), negative regulation of BMP signaling (4 proteins), and synaptic vesicle priming. These changes imply that Ejiao reverses transcriptional repression, stabilizes microtubules, normalizes calcium signaling, and attenuates overactive BMP activity.

KEGG pathway analysis (Figure 8C,D) revealed that in the M_vs_N comparison, steroid hormone biosynthesis was the most dramatically altered pathway (8 proteins, mostly down regulated), along with disturbances in cAMP, calcium, and cellular senescence pathways. Notably, the PI3K-Akt, MAPK, and mTOR pathways were also enriched. In contrast, Ejiao treatment prominently reversed these alterations, with the calcium signaling pathway becoming the most significantly enriched (8 proteins), followed by cAMP and cGMP-PKG pathways. The Wnt signaling pathway (5 proteins) was enriched, with up regulation of β catenin, suggesting involvement rather than demonstrated activation. Importantly, steroid hormone biosynthesis remained enriched but with fewer down regulated proteins, and key enzymes CYP17A1 and CYP19A1 were up regulated. These results indicate that Ejiao is associated with modulation of calcium/cAMP/cGMP-PKG/Wnt axes, while mitigating the aberrant PI3K-Akt/mTOR and senescence signals.

#### 3.6.3. Protein–Protein Interaction Network Identifies Hub Proteins in TGF-β and Wnt Modules

**Figure 9:**
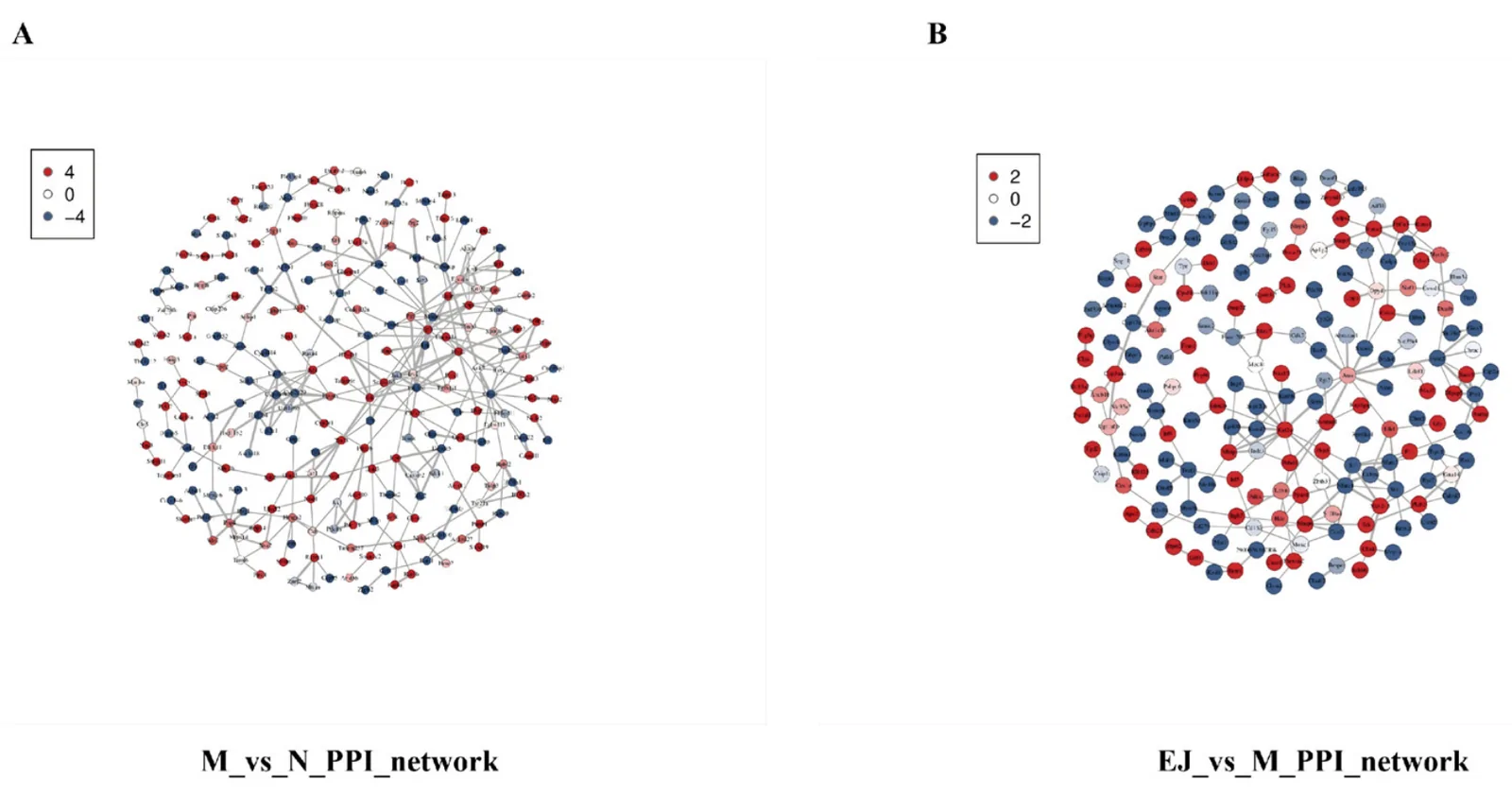
Protein–Protein Interaction (PPI) Network Analysis. (**A**) M_vs_N_PPI_network. (**B**) EJ_vs_M_PPI_network. [*n* = 3 biological replicates per group (each pool = 3 mice; 9 mice contributed per group)].

Protein–protein interaction (PPI) networks were constructed to identify hub proteins (Figure 9A,B). In the M vs. N network, proteins with high connectivity (degree ≥ 8) included BMP2, BMP4, MMP14, MMP2, MMP13, FGFR1, CTNNB1, NOTCH1, FN1, RAC1, SPP1, and COL1A1 (Table 2). These proteins formed an interconnected module centered on BMP/Wnt signaling and extracellular matrix (ECM) remodeling. In the EJ vs. M network, the hub proteins were TGFBR2, SMAD3, MMP14, and SMAD2 (Table 3). This network was organized around the TGF-β/Smad signaling pathway, with TGFBR2 and SMAD3 upregulated and SMAD2 downregulated. Notably, MMP14 exhibited opposite regulation (down in M_vs_N, up in EJ_vs_M), suggesting its role as a bidirectional switch in ECM remodeling during POI progression.

## 4. Discussion

In the present study, we established a POI mouse model using tripterygium glycosides and systematically evaluated the therapeutic effects of Ejiao. Our results demonstrate that Ejiao effectively restored the ovarian index and tended to improve the uterine index (non-significant), corrected estrous-cycle disturbance and partially corrected the endocrine profile (E2, AMH, GnRH), ameliorates follicular development, and alleviates bone microarchitectural deterioration in TG-induced POI mice.

Consistent with our findings, similar trends have been reported in other intervention studies: hyperin significantly increased the gonadal index in TG-induced POI mice [49]; melatonin improved ovarian and uterine mass and indices in TG-exposed mice [25]; edible bird’s nest increased the ovarian index in TG-induced POI rats [50]; and Bushen–Culuan decoction resulted in a significantly higher ovarian index than that of the model group [51]. These interventions also improved follicular development—Bushen–Culuan decoction reduced the number of atretic follicles while increasing primordial follicles in TG-induced POI mice [52], and Yijing decoction reduced follicular atresia and maintained nearly normal ovarian size in triptolide-induced POI rats [53]. Collectively, these findings support the notion that TG impairs ovarian and uterine development and that natural compounds, including Ejiao, can counteract this damage by improving ovarian indices and follicular development.

POI patients typically have low estrogen and high gonadotropin levels. Our model mice showed the same picture: low E2 and AMH, high FSH, LH, GnRH and PRL. Those changes match what others have reported in POI patients and in chemically induced models [24,54]. After Ejiao treatment, E2 and AMH went up, and GnRH came down—in a dose-dependent way. Similar hormonal improvements have been reported for other herbal formulas in POI models: Kuntai capsules raised E2 and AMH and lowered FSH in cyclophosphamide-induced POI rats [55]; Erxian decoction did the same in other POI models [56]; and Zuogui pill also increased AMH and E2 while reducing FSH [57]. Together, these observations suggest that multiple traditional Chinese medicine (TCM) interventions restore hormone balance in POI, most likely through regulation of the hypothalamic–pituitary–ovarian (HPO) axis.

The fact that Ejiao increased AMH is worth highlighting. AMH is produced by granulosa cells in small growing follicles, and it is one of the best markers we have for ovarian reserve [58]. Raising AMH suggests that Ejiao helps protect the pool of growing follicles and keeps granulosa cells functioning better.

From a compositional perspective, several nutritional supplements and traditional preparations evaluated in POI models—edible bird’s nest, Bushen–Culuan and Yijing decoctions, hyperin, and melatonin—share bioactive classes such as proteins/peptides, polysaccharides, and antioxidant constituents. Ejiao’s distinguishing feature is its collagen/gelatin peptide profile. We do not intend to imply that these interventions share identical mechanisms, and a compositional comparison across them represents a valuable direction for future research.

At the proteomic level, 313 differentially expressed proteins (DEPs) were identified between the Ejiao-treated (EJ) and model (M) groups. KEGG enrichment analysis indicated that these DEPs were associated with calcium signaling, cAMP, cGMP–PKG, and TGF-β/Smad pathways. These observations suggest the involvement of multiple signaling pathways linked to Ejiao intervention in the POI model, although the underlying mechanisms remain to be established by functional validation.

Calcium signaling matters for oocyte maturation, fertilization, and steroid production in granulosa cells [11,59]. Calcium also affects the cAMP system; without enough calcium, granulosa cells do not accumulate cAMP well when LH stimulates them [11]. cAMP and cGMP-PKG are major routes through which gonadotropins act on the ovary. The fact that Ejiao affects all three pathways suggests it helps restore the basic signaling machinery that controls follicle development and hormone synthesis.

TGF-β/Smad signaling came out as a key node in our PPI network. TGFBR2 (degree 15, upregulated) and SMAD3 (degree 11, upregulated) were hub proteins in the Ejiao-vs-model network. The TGF-β superfamily is known to regulate follicle activation, the transition from dormant to growing follicles, granulosa cell proliferation, and steroid production [16]. Polymorphisms in TGF-β pathway genes—including TGF-β and BMP15—have been linked to a higher risk of POI, indicating that this pathway helps determine ovarian reserve [60]. BMP2 and BMP4 were also upregulated in our model (M vs. N). It is known that oocyte-secreted factors such as BMP15 and GDF9, both members of the TGF-β superfamily, play essential roles in follicular development, and mutations in those genes are associated with POI [61]. Ejiao upregulated TGFBR2 and SMAD3 while partially reversing the abnormal BMP2/4 changes—so it may help rebalance TGF-β superfamily signaling rather than simply turning it on or off.

MMP14 showed opposite regulation in the two comparisons. It was downregulated in POI (M vs. N) but came back up after Ejiao treatment (EJ vs. M). MMP14 (MT1-MMP) is a key enzyme for ECM remodeling, which is needed for follicle rupture and ovulation [62]. In primates, hCG induces MMP14 and MMP16 in the preovulatory follicle, and this is conserved across species [22]. Problems with ECM remodeling can lead to ovarian fibrosis, and fibrosis has been suggested to contribute to POI pathophysiology; stromal fibrosis correlates with worsening ovarian function [63,64]. Restoring MMP14 expression might therefore help normalize ECM remodeling during follicular development and ovulation, and possibly reduce fibrosis that damages ovarian reserve.

Wnt/β-catenin signaling also appeared in our data. CTNNB1 (β-catenin) was downregulated in POI mice. Wnt signaling helps maintain the primordial follicle pool and regulates ovarian stroma [65]. Loss of β-catenin has been shown to accelerate follicular atresia [66].

Estrogen deficiency from POI often leads to osteoporosis, with lower bone mass, altered microarchitecture, and higher fracture risk [42]. Our model mice had lower BMD, fewer trabeculae (Tb.N, Tb.Th), and more separation (Tb.Sp). Ejiao treatment improved all these measures. The RANKL/OPG/RANK system controls bone resorption. When estrogen drops, OPG activity goes down and RANKL expression goes up, shifting the balance toward more bone breakdown. We found that Ejiao raised OPG and lowered RANKL, which reduced the RANKL/OPG ratio. Others have reported similar findings: Ejiao-containing formulas promote osteoblast activity and increase OPG while decreasing RANKL [47,67]. One study also showed that Ejiao suppresses elevated bone turnover and prevents body composition changes in ovariectomized rats, mainly through antioxidant effects [67]. Interestingly, Ejiao has been shown to upregulate TGF-β1 mRNA in osteoblasts [47], which fits with our observation that Ejiao modulates TGF-β signaling in the ovary—a possible sign of a systemic effect.

Notably, the proportion of CD4^+^CD25^+^Foxp3^+^ Tregs increased significantly with the low dose but not the high dose. This non-monotonic response, which was not observed for other outcomes, may reflect bell-shaped immunomodulation or counter-regulatory feedback at higher doses, and warrants further investigation; it indicates that the immunomodulatory effects of Ejiao may not follow a simple dose–response relationship.

Taken together, Ejiao seems to work through several routes: (1) adjusting hormone levels via the HPO axis-raising E2 and AMH while lowering GnRH; (2) being associated with protective pathways in the ovary, including calcium signaling, cAMP/cGMP-PKG, and TGF-β/Smad; (3) bringing MMP14 back toward normal to support ECM remodeling and potentially limit fibrosis; (4) affecting Wnt/β-catenin signaling; and (5) regulating the RANKL/OPG axis to reduce bone loss. This multi-target profile makes sense for a complex condition like POI, where single-target drugs often have limitations.

This study has several limitations: (1) the POI model was chemically induced by tripterygium glycosides and may not fully recapitulate idiopathic human POI; (2) the estrous-cycle monitoring was likewise of short duration; (3) fertility and pregnancy outcomes were not assessed; (4) no hormone-replacement-therapy comparator group was included; (5) As a naturally derived product, the composition of Ejiao may vary across batches and sources; only one standardized batch of Ejiao was used throughout this study; (6) GnRH is secreted in a pulsatile manner and mainly enters the hypophyseal portal circulation, so the single terminal peripheral serum GnRH measurement used here can only serve as a surrogate indicator and cannot fully reflect the pulsatile pattern of hypothalamic GnRH release; and (7) the proteomic findings remain correlative—Western blot or qPCR validation of the candidate hub proteins (TGFBR2, SMAD3, MMP14, and β-catenin) has not yet been performed, and enrichment of the TGF-β/Smad and Wnt pathways indicates association rather than demonstrated causal activation. Orthogonal validation is planned in future work. These constraints should be considered when interpreting the results and their generalizability.

## 5. Conclusions

In a mouse model of tripterygium glycoside-induced premature ovarian insufficiency (POI), oral administration of Ejiao restored the ovarian index and tended to improve the uterine index (non-significant), corrected estrous-cycle disturbance, partially corrected serum hormone profiles, supported follicular development, and attenuated bone loss. Quantitative proteomics identified 313 differentially expressed proteins between Ejiao-treated and model groups, with enrichment in calcium signaling, cAMP, cGMP-PKG, and TGF-β/Smad pathways. TGFBR2 and SMAD3 emerged as hub proteins in the Ejiao-responsive network. MMP14 was dysregulated in POI but modulated by Ejiao, suggesting a role in extracellular matrix remodeling. Additionally, Ejiao modulated the RANKL/OPG axis to counteract POI-induced osteoporosis.

These findings provide the first comprehensive proteomic insight into the molecular correlates associated with Ejiao’s amelioration of POI, supporting its potential as a natural bioactive nutritional intervention for POI and its associated complications. The multi-pathway regulatory profile of Ejiao warrants further preclinical and clinical investigation; comprehensive safety evaluation and a head-to-head comparison with hormone replacement therapy were not performed in this study.

## Figures and Tables

**Table 1:** Estrous cycle length and grading of estrous cycle dysfunction in POI mice. (*n* = 6 mice per group).

Group	Estrous Cycle (Days)	Grade I	Grade II	Grade III	Grade IV
Negative control group	9.3 ± 0.52	6	0	0	0
Model group	10.8 ± 0.29 **	0	0	4	2
EJL group	9.8 ± 0.96	3	2	1	0
EJH group	8.5 ± 0.50 ^##^	3	1	2	0

Note: Compared with negative control group, ** *p* < 0.01; compared with model group, ## *p* < 0.01.

**Table 2:** Proteins with degree ≥ 8 in the M_vs_N network. [*n* = 3 biological replicates per group (each pool = 3 mice; 9 mice contributed per group).]

Accession	Predicted Gene	Degree	Regulation	Main Function
P05017	BMP2	14	up	Bone morphogenetic protein, follicle development
P41245	MMP14	14	down	Membrane-type matrix metalloproteinase, ECM remodeling
A2AAJ9	FGFR1	13	down	FGF receptor, granulosa cell proliferation
O35904	CTNNB1 (β-catenin)	13	down	Core component of Wnt signaling
Q99JW5	MMP2	12	up	Matrix metalloproteinase 2
B1ASP2	NOTCH1	10	down	Notch receptor
P35569	BMP4	10	up	Bone morphogenetic protein 4
Q64458	FN1	9	down	Fibronectin, extracellular matrix
P35991	RAC1	8	up	Small GTPase, cytoskeletal regulation
Q02956	(CTNNB1-related)	8	up	Wnt signaling
P10923	SPP1	8	up	Osteopontin, immune and matrix remodeling
P30561	COL1A1	8	up	Collagen type I alpha 1
Q9EQW4	MMP13	8	down	Matrix metalloproteinase 13

**Table 3:** Proteins with degree ≥ 8 in the EJ_vs_M network. [*n* = 3 biological replicates per group (each pool = 3 mice; 9 mice contributed per group).]

Accession	Predicted Gene	Degree	Regulation	Main Function
Q9JHD2	TGFBR2	15	up	TGF-β receptor, granulosa cell apoptosis
Q62388	SMAD3	11	up	TGF-β signal transducer
P41245	MMP14	8	up	Matrix metalloproteinase 14
Q9JM73	SMAD2	8	down	TGF-β signal transducer

## Data Availability

The raw proteomic data cannot be deposited in public repositories due to the limited data retention policy of our contracted proteomics service provider. All other raw data supporting the findings of this study are available within the article and its Supplementary Materials. Further inquiries related to the datasets can be directed to the corresponding author upon reasonable request.

## Supplementary Materials

The following supporting information can be downloaded at: https://www.mdpi.com/article/10.3390/nu18213595/s1, File S1: Proteomics analysis; Excel S1: effect sizes and 95% confidence intervals; Excel S2: ndividual longitudinal cycle data; File S2: representative flow-cytometry gating plots.
