# Supplementary material for: Integrative Proteomics Reveals That Ejiao Alleviates Premature Ovarian Insufficiency and Restores Bone Metabolism in Mice
Source: Nutrients. 2026 Oct 29;18(21):3595. doi: 10.3390/nu18213595 (PMC13764925; doi:10.3390/nu18213595)
Supplement: Supplementary file 1 [file nutrients-18-03595-s001.zip › File S1 Proteomics analysis/WPCNA/1.软阈值选择/Fig2_PickSoftpower.pdf]

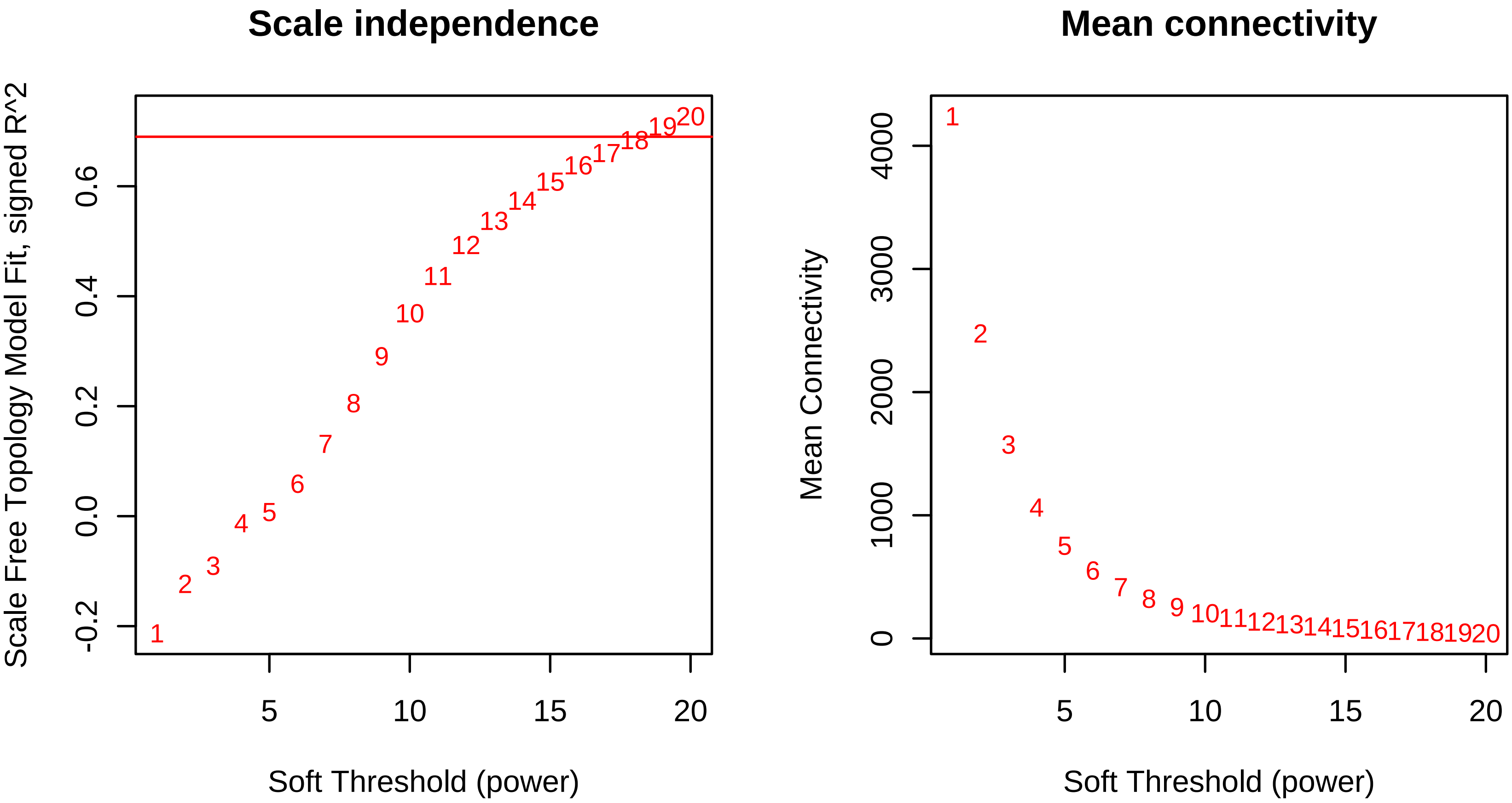
Scale independence
Scale Free Topology Model Fit, signed R^2
-0.2
0.0
0.2
0.4
0.6
1
2
3
4
5
6
7
8
9
10
11
12
13
14
15
16
17
18
19
20
5
10
15
20
Soft Threshold (power)
Mean connectivity
Mean Connectivity
0
1000
2000
3000
4000
1
2
3
4
5
6
7
8
9
10
11
12
13
14
15
16
17
18
19
20
5
10
15
20
Soft Threshold (power)
