# Supplementary material for: Integrative Proteomics Reveals That Ejiao Alleviates Premature Ovarian Insufficiency and Restores Bone Metabolism in Mice
Source: Nutrients. 2026 Oct 29;18(21):3595. doi: 10.3390/nu18213595 (PMC13764925; doi:10.3390/nu18213595)
Supplement: Supplementary file 1 [file nutrients-18-03595-s001.zip › File S1 Proteomics analysis/WPCNA/3.模块特征及表达模式/Fig5_ModuleSamplesCorrelation.pdf]

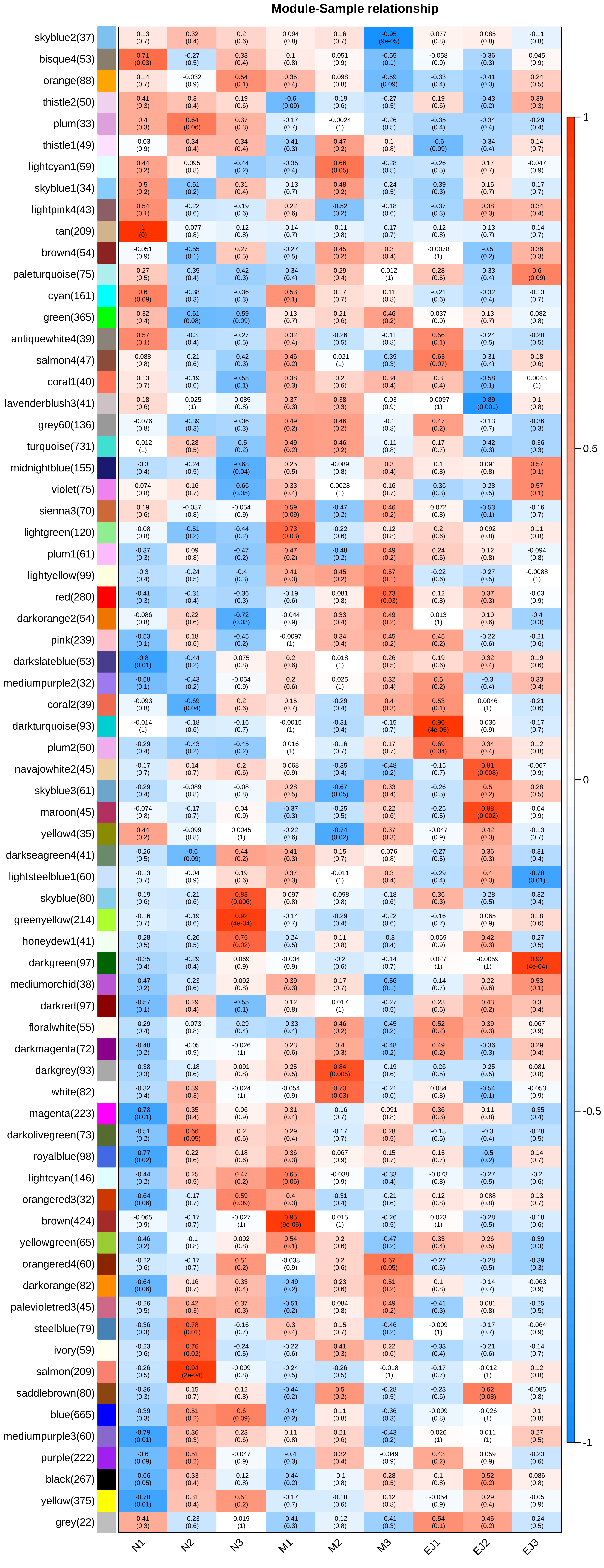
Module-Sample relationship
skyblue2(37)
bisque4(53)
orange(88)
thistle2(50)
plum(33)
thistle1(49)
lightcyan1(59)
skyblue1(34)
lightpink4(43)
tan(209)
brown4(54)
paleturquoise(75)
cyan(161)
green(365)
antiquewhite4(39)
salmon4(47)
coral1(40)
lavenderblush3(41)
grey60(136)
turquoise(731)
midnightblue(155)
violet(75)
sienna3(70)
lightgreen(120)
plum1(61)
lightyellow(99)
red(280)
darkorange2(54)
pink(239)
darkslateblue(53)
mediumpurple2(32)
coral2(39)
darkturquoise(93)
plum2(50)
navajowhite2(45)
skyblue3(61)
maroon(45)
yellow4(35)
darkseagreen4(41)
lightsteelblue1(60)
skyblue(80)
greenyellow(214)
honeydew1(41)
darkgreen(97)
mediumorchid(38)
darkred(97)
floralwhite(55)
darkmagenta(72)
darkgrey(93)
white(82)
magenta(223)
darkolivegreen(73)
royalblue(98)
lightcyan(146)
orangered3(32)
brown(424)
yellowgreen(65)
orangered4(60)
darkorange(82)
palevioletred3(45)
steelblue(79)
ivory(59)
salmon(209)
saddlebrown(80)
blue(665)
mediumpurple3(60)
purple(222)
black(267)
yellow(375)
grey(22)
N1
N2
N3
M1
M2
M3
EJ1
EJ2
EJ3
1
0.5
0
-0.5
-1
