# Supplementary material for: Integrative Proteomics Reveals That Ejiao Alleviates Premature Ovarian Insufficiency and Restores Bone Metabolism in Mice
Source: Nutrients. 2026 Oct 29;18(21):3595. doi: 10.3390/nu18213595 (PMC13764925; doi:10.3390/nu18213595)
Supplement: Supplementary file 1 [file nutrients-18-03595-s001.zip › File S1 Proteomics analysis/WPCNA/3.模块特征及表达模式/Fig7_ProteinWithSample.pdf]

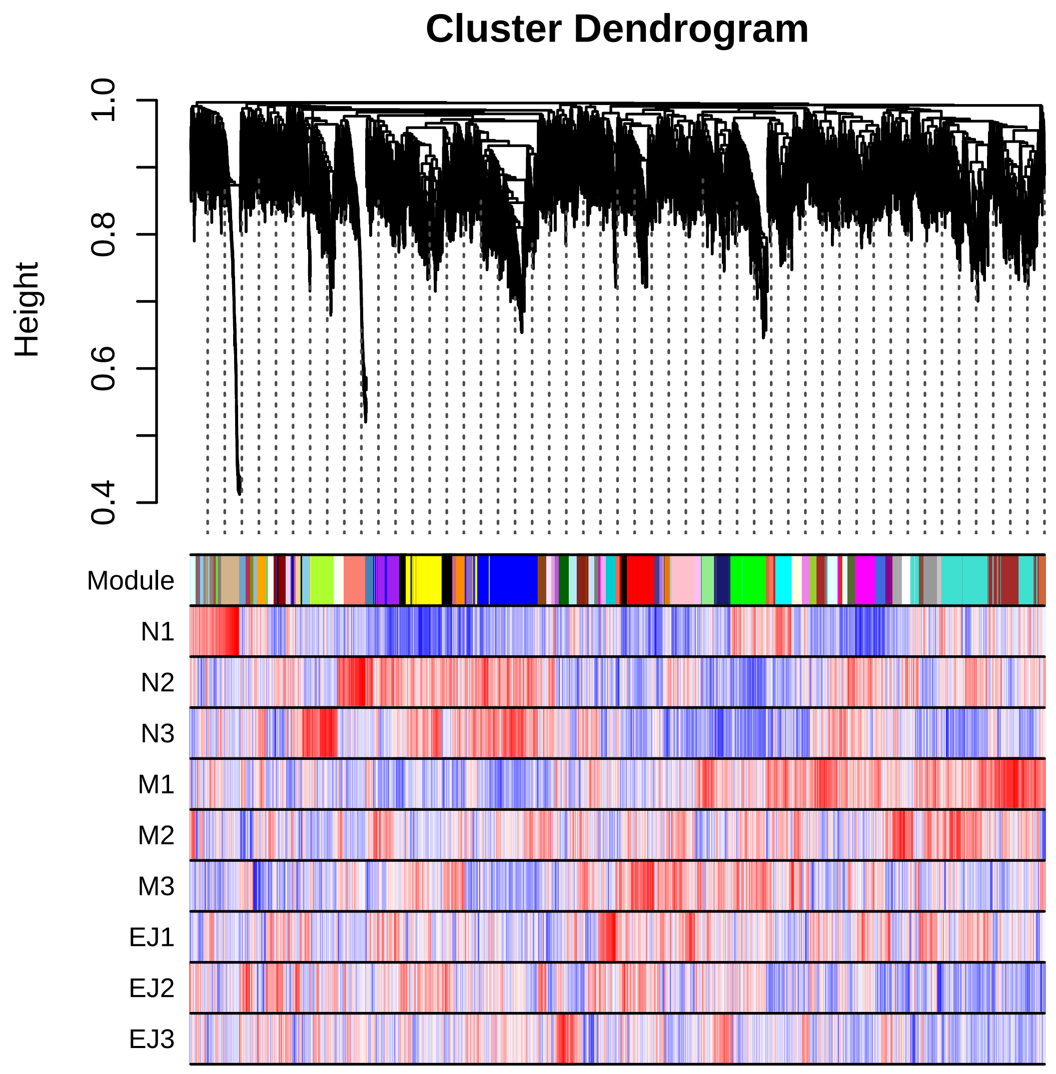
Cluster Dendrogram
Height
1.0
0.8
0.6
0.4
Module
N1
N2
N3
M1
M2
M3
EJ1
EJ2
EJ3
