# Supplementary material for: Integrative Proteomics Reveals That Ejiao Alleviates Premature Ovarian Insufficiency and Restores Bone Metabolism in Mice
Source: Nutrients. 2026 Oct 29;18(21):3595. doi: 10.3390/nu18213595 (PMC13764925; doi:10.3390/nu18213595)
Supplement: Supplementary file 1 [file nutrients-18-03595-s001.zip › File S2 representative flow-cytometry gating plots.pdf]

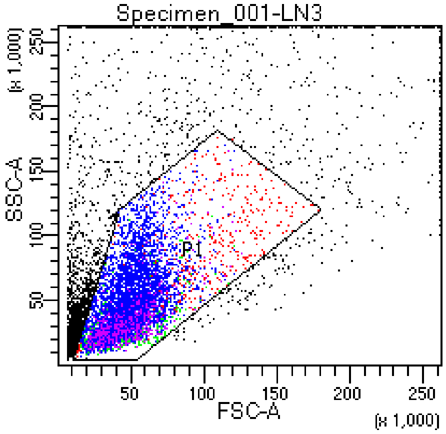

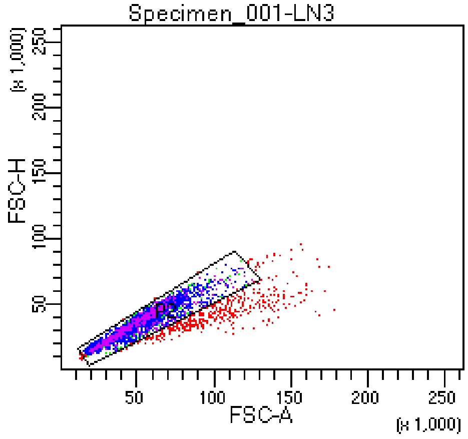

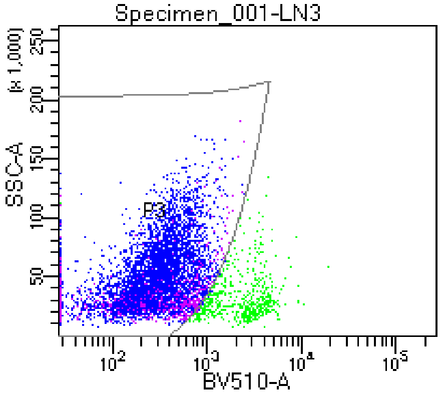

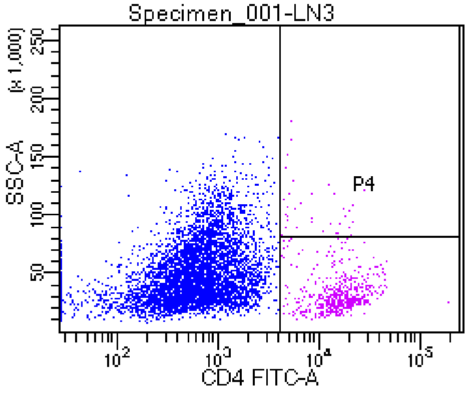

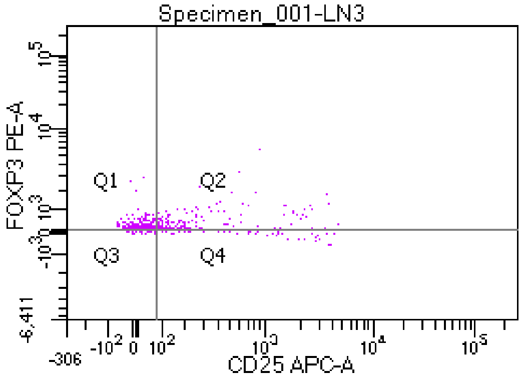

Tube: LN3

Population	#Events	%Parent
All Events	10,000	####
P1	6,487	64.9
P2	6,027	92.9
P3	5,472	90.8
P4	509	9.3
Q1	234	46.0
Q2	149	29.3
Q3	69	13.6
Q4	57	11.2

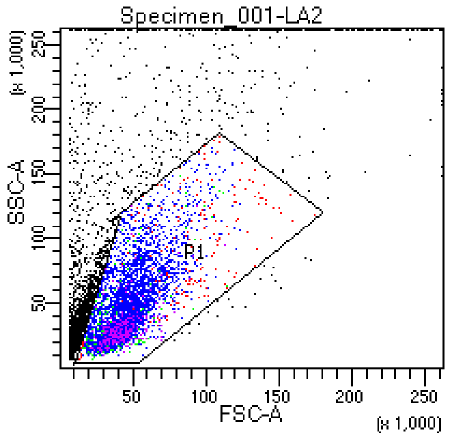

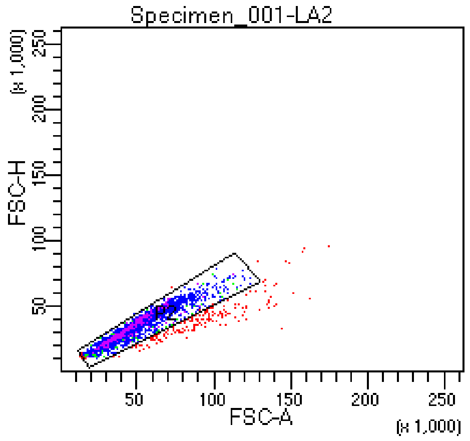

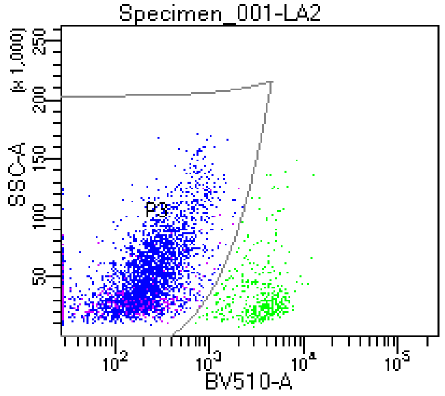

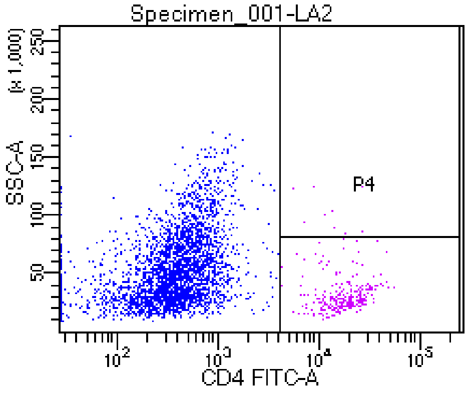

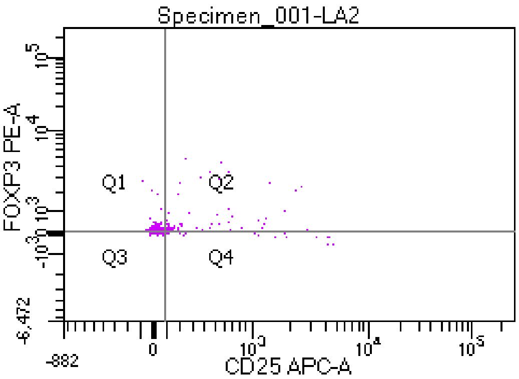

Tube: LA2

Population	#Events	%Parent
All Events	6,970	####
P1	4,055	58.2
P2	3,851	95.0
P3	3,396	88.2
P4	263	7.7
Q1	118	44.9
Q2	57	21.7
Q3	65	24.7
Q4	23	8.7
